# Antithrombotic therapy in diabetes: which, when, and for how long?

**DOI:** 10.1093/eurheartj/ehab128

**Published:** 2021-03-25

**Authors:** Ramzi A Ajjan, Noppadol Kietsiriroje, Lina Badimon, Gemma Vilahur, Diana A Gorog, Dominick J Angiolillo, David A Russell, Bianca Rocca, Robert F Storey

**Affiliations:** The LIGHT Laboratories, Leeds Institute of Cardiovascular and Metabolic Medicine, University of Leeds, Leeds LS2 7JT, UK; The LIGHT Laboratories, Leeds Institute of Cardiovascular and Metabolic Medicine, University of Leeds, Leeds LS2 7JT, UK; Endocrinology and Metabolism Unit, Internal Medicine Department, Faculty of Medicine, Prince of Songkla University, Songkhla 90110, Thailand; Cardiovascular Program ICCC, Research Institute Hospital de la Santa Creu i Sant Pau, IIB-Sant Pau, Sant Antoni M. Claret 167, 08025 Barcelona, Spain; Centro de Investigación Biomédica en Red Cardiovascular (CIBERCV), Instituto de Salud Carlos III, Sant Antoni M. Claret 167, 08025 Barcelona, Spain; Cardiovascular Research Chair, Universidad Autónoma Barcelona (UAB), Sant Antoni M. Claret 167, 08025 Barcelona, Spain; Cardiovascular Program ICCC, Research Institute Hospital de la Santa Creu i Sant Pau, IIB-Sant Pau, Sant Antoni M. Claret 167, 08025 Barcelona, Spain; Centro de Investigación Biomédica en Red Cardiovascular (CIBERCV), Instituto de Salud Carlos III, Sant Antoni M. Claret 167, 08025 Barcelona, Spain; University of Hertfordshire, College Lane Campus Hatfield, Hertfordshire AL10 9AB, UK; National Heart and Lung Institute, Guy Scadding Building, Dovehouse St, London SW3 6LY, UK; Division of Cardiology, University of Florida College of Medicine – Jacksonville, 655 West, 8th Street, Jacksonville, FL 32209, USA; The LIGHT Laboratories, Leeds Institute of Cardiovascular and Metabolic Medicine, University of Leeds, Leeds LS2 7JT, UK; Leeds Vascular Institute, Leeds General Infirmary, Great George Street, Leeds LS1 3EX, UK; Institute of Pharmacology, Catholic University School of Medicine, Rome, Italy; Department of Infection, Immunity and Cardiovascular Disease, University of Sheffield, Beech Hill Road, Sheffield S10 2RX, UK

**Keywords:** Diabetes, Cardiovascular, Cerebrovascular, Peripheral artery disease, Antithrombotic, Antiplatelet

## Abstract

Cardiovascular disease remains the main cause of mortality in individuals with diabetes mellitus (DM) and also results in significant morbidity. Premature and more aggressive atherosclerotic disease, coupled with an enhanced thrombotic environment, contributes to the high vascular risk in individuals with DM. This prothrombotic milieu is due to increased platelet activity together with impaired fibrinolysis secondary to quantitative and qualitative changes in coagulation factors. However, management strategies to reduce thrombosis risk remain largely similar in individuals with and without DM. The current review covers the latest in the field of antithrombotic management in DM. The role of primary vascular prevention is discussed together with options for secondary prevention following an ischaemic event in different clinical scenarios including coronary, cerebrovascular, and peripheral artery diseases. Antiplatelet therapy combinations as well as combination of antiplatelet and anticoagulant agents are examined in both the acute phase and long term, including management of individuals with sinus rhythm and those with atrial fibrillation. The difficulties in tailoring therapy according to the variable atherothrombotic risk in different individuals are emphasized, in addition to the varying risk within an individual secondary to DM duration, presence of complications and predisposition to bleeding events. This review provides the reader with an up-to-date guide for antithrombotic management of individuals with DM and highlights gaps in knowledge that represent areas for future research, aiming to improve clinical outcome in this high-risk population.

## Graphical Abstract

**Figure:**
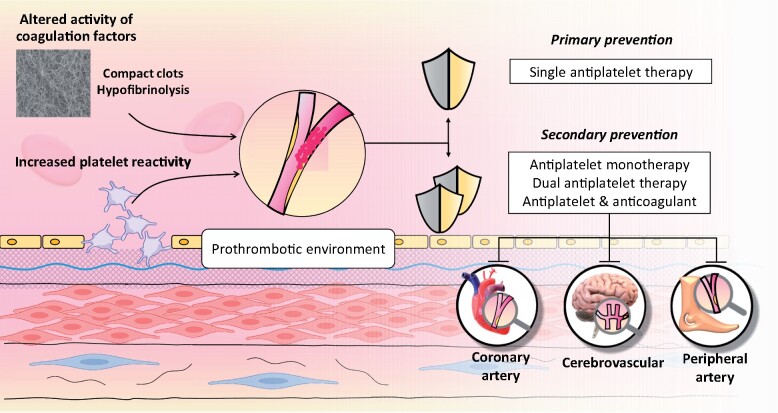
Atherothrombosis in diabetes.

## Introduction

Despite advances in therapy, a diagnosis of diabetes mellitus (DM) is associated with increased morbidity and reduced lifespan, mainly due to vascular complications.^1–3^ Premature and more severe vascular disease, as well as a prothrombotic environment, represent key mechanisms for adverse vascular outcomes in this population.^4^ The prothrombotic milieu develops secondary to increased platelet reactivity coupled with hypofibrinolysis.^5^ ^,^ ^6^

Current treatment strategies to improve vascular outcomes in individuals with DM are focused on revascularization of acute atherothrombotic occlusions, where possible, together with early introduction of antithrombotic therapies, usually by inhibiting platelet function. This continues long-term coupled with multifactorial therapy targeting hypertension, dyslipidaemia and dysglycaemia in order to limit the progression of vascular pathology.

In this review, we discuss the latest in antithrombotic therapies for the management of coronary artery disease (CAD), cerebrovascular disease, and peripheral artery disease (PAD) in DM, covering therapies for primary prevention, acute vascular occlusion and long-term secondary prevention. Special emphasis is placed on the benefits and risks of antithrombotic therapy combinations, with the overall aim of providing the reader with an up-to-date guide for antithrombotic management in DM. Search strategy is detailed in the Supplementary material online, *S1*. 

## The thrombotic environment in diabetes

**Figure 1:**
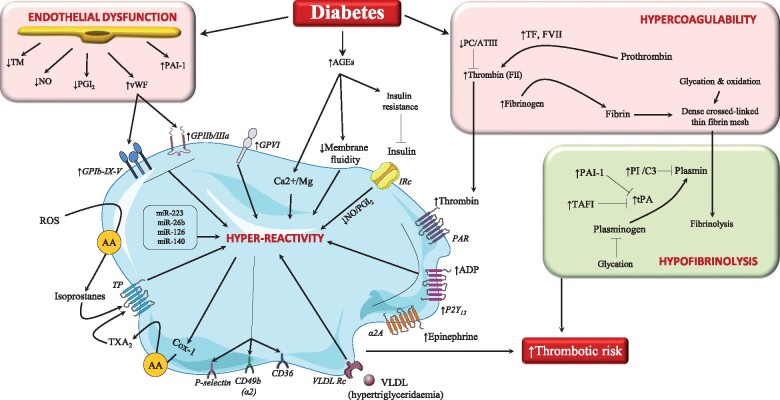
Diabetes enhances the risk of thrombosis. Diabetes induces endothelial dysfunction with subsequent decline in the expression/release of molecules that can reduce platelet activation and associated thrombus formation. At a platelet level, there are several mechanisms by which diabetes could enhance platelet susceptibility to activation including: (i) a higher abundance of advanced glycation end-products (AGEs) which induces insulin resistance and alters membrane fluidity; (ii) an enhanced oxidative stress which leads to the formation of isoprostanes which in turn induce platelet activation by interacting with the thromboxane receptor (TP); (iii) a higher production of thromboxane (TXA_2_); and (iv) an increased expression of multiple platelet activation receptors and a higher reactivity to several platelet agonists. As for coagulation, diabetes is associated with a higher amount of tissue factor (TF), thrombin (factor II), and fibrinogen production, which, in concurrence with lower anticoagulant proteins [protein C-antithrombin III complex (PC/ATIII)], favours the formation of the fibrin mesh, which undergoes glycation and oxidative modifications, becoming more dense and resistant to fibrinolysis. Diabetes is also associated with a hypofibrinolytic state characterized by higher abundance of inhibitors of tissue plasminogen activator (tPA) such as plasminogen activator inhibitor-1 (PAI-1) and thrombin-activatable fibrinolysis inhibitor (TAFI), and increased incorporation of antifibrinolytic proteins into the clot [plasmin inhibitor (PI) and complement 3 (C3)] which collectively reduce the efficiency of fibrinolysis. AA: arachidonic acid; ADP: adenosine diphosphate; Cox: cyclooxygenase; GP: glycoprotein; IRc: insulin receptors; miR: microRNAs; NO: nitric oxide; PAR: protease-activated receptor; PGI_2_: prostacyclin; ROS: reactive oxygen species; TM: thrombomodulin; VLDL: very-low-density lipoprotein; vWF: von Willebrand factor.

Individuals with DM are prone to both arterial and venous thrombosis.^7^ DM is characterized by multiple pathological processes, including hyperglycaemia, chronic inflammation, oxidative stress, and associated metabolic conditions, that damage the endothelium and increase platelet reactivity, resulting in a prothrombotic environment. Endothelial dysfunction is a consistent finding in DM patients and contributes to the prothrombotic shift (*Figure 1*).^4^

An array of mechanisms operate in platelets to enhance their reactivity.^8^ Hyperglycaemia is associated with higher expression of platelet receptors, including glycoprotein (GP) Ib*α*, GPIIb/IIIa and P2Y_12_,^9^ reduced platelet membrane fluidity secondary to increased glycation, higher thromboxane (TX) A_2_ synthesis together with increased platelet activation markers.^10–12^ Diabetes-associated oxidative stress also increases production of F2-isoprostanes.^13^ TXA_2_ and F2-isoprostanes, in turn, activate the thromboxane receptors and amplify platelet activation (*Figure 1*).^14^ Platelet hyper-reactivity also results from diminished sensitivity to the inhibitory agents prostacyclin, nitric oxide, and insulin,^15–17^ and from changes in platelet content of miRNAs known to regulate platelet function (miR-223, miR-26b, miR-126, miR-140).^18^ ^,^ ^19^ Moreover, the imbalance in intraplatelet magnesium and calcium homeostasis renders platelets more sensitive to epinephrine, adenosine diphosphate (ADP), and thrombin.^20^ DM is also characterized by accelerated platelet turnover, as evidenced by release of more reactive, reticulated platelets^21^ ^,^ ^22^ that display a reduced response to antiplatelet agents.^23^ Finally, platelets from DM patients more easily externalize phosphatidylserine in the outer platelet membrane, thereby providing a better surface for the assembly of clotting factors and tissue factor activation.^21^ ^,^ ^22^

Other associated metabolic conditions like obesity, dyslipidaemia, and systemic inflammation also contribute to thrombosis risk.^6^ ^,^ ^24^ Circulating inflammatory molecules [tumour necrosis factor-α, interleukin (IL)-1 and IL-6, selectin, soluble CD40 ligand],^17^ ^,^ ^25^ besides enhancing platelet reactivity, favour a hypercoagulable environment. Furthermore, bone marrow transplants that created chimeras of normal rats with bone marrow cells from diabetic rats resulted in a prothrombotic phenotype similar to the donor animals, indicating the imprinting effects of DM on haematopoietic cells.^22^

Several prothrombotic alterations in the coagulation-fibrinolytic system also occur in DM,^26^ including increased levels of tissue factor, prothrombin, factor VII and fibrinogen coupled with impaired anticoagulant and fibrinolytic activity (*Figure 1*).^27^ Diabetic thrombi display compact fibrin networks with densely-packed thin fibres that are resistant to fibrinolysis.^6^ ^,^ ^26^ Furthermore, hyperglycaemia induces qualitative changes in plasminogen, hindering its fibrinolytic activity.^28^ Concomitantly, elevated levels of anti-fibrinolytic proteins (plasminogen activator inhibitor-1 and thrombin-activatable fibrinolysis inhibitor),^29^ along with increased incorporation of anti-fibrinolytic proteins (complement C3 and plasmin inhibitor) into the clot further compromise fibrinolysis.^6^ Despite this prothrombotic environment, DM patients have a paradoxical increased risk of bleeding, particularly following an acute coronary syndrome (ACS).^30^ However, data on stable patients are less clear: the dual antiplatelet therapy (DAPT) study (detailed below under ‘Secondary Prevention’) reported no increase in long-term moderate or severe bleeding events in those with DM, possibly related to excluding those with bleeding events in the first 12 months, while increased risk was documented in the REACH and CORONOR registries.^31^ ^,^ ^32^ The exact mechanisms for the increase in both thrombosis and bleeding risk in some diabetes patients are not fully understood, although renal complications may have a role.^33^ Also, chronic activation of platelet and coagulation proteins may ‘exhaust’ the system in some DM patients, thus increasing bleeding risk.^34^

## Antithrombotic targets

For decades, the two main antithrombotic targets have been platelet TXA_2_ production and platelet P2Y_12_ receptor activation.

### Aspirin

Low-dose aspirin irreversibly inhibits platelet cyclooxygenase-1 enzyme, preventing the conversion of arachidonic acid into bioactive prostanoid TXA_2_.^35^ Given the short half-life of aspirin and increased platelet turnover in DM, a proportion of platelets may escape 24-h inhibition by once-daily aspirin, which can be re-established by twice-daily dosing.^23^ ^,^ ^36^

### P2Y_12_ receptor antagonists

ADP-stimulated effects on platelets are mediated primarily by Gi-coupled P2Y_12_ receptor activation, leading to persistent platelet aggregation, whereas P2Y_1_ is responsible for an initial weak, transient phase of platelet aggregation.^37^ There are two main classes of orally administered P2Y_12_ inhibitors: thienopyridines (ticlopidine, clopidogrel, and prasugrel) and non-thienopyridine agents (ticagrelor).^38^ Thienopyridines require conversion to an active metabolite that acts irreversibly. Ticlopidine is no longer marketed in many countries due to safety concerns.^38^ Ticagrelor is a direct-acting cyclopentyltriazolopyrimidine that requires no metabolism and binds reversibly to the P2Y_12_ receptor.^38^

### Other antithrombotic approaches

Warfarin and other vitamin K antagonists (VKAs) require regular monitoring and this, together with high bleeding risk when combined with antiplatelet therapy, prevented widespread use.^39^ More modern approaches include modulation of thrombin activity either by blocking protease-activated receptor-1 on platelet membrane (vorapaxar)^40^ or by directly inhibiting protein function (dabigatran).^41^ Other non-VKA oral anticoagulants (NOAC) include inhibitors of activated factor Xa (apixaban, rivaroxaban, and edoxaban).^41^  Supplementary material online, *Table S1* provides a summary of the main antithrombotic agents.

## Antiplatelet therapy for primary prevention of ischaemic events

Primary prevention is defined as offering therapy to individuals without a history of a vascular ischaemic event. In the largest individual data meta-analysis of primary prevention trials (*n* = 95 000 individuals), aspirin use in DM was associated with a non-significant 12% relative risk reduction (RRR) of major adverse cardiac events (MACE), from 1.87% to 1.63% per year [hazard ratio (HR) 0.88 (0.67–1.15); *Table 1*].^42^ Although non-significant, this benefit was comparable to non-DM individuals, in whom aspirin reduced yearly MACE from 0.57% to 0.51% [HR 0.88 (0.82–0.94); *P* = 0.0001].^42^ Aspirin was associated with an increase in extracranial, mainly gastrointestinal, bleeding in both non-DM and DM populations (*P* = 0.20 for heterogeneity; *Table 1*).^42^ Following this meta-analysis, the Japanese Primary Prevention of Atherosclerosis With Aspirin for Diabetes (JPAD) (*n* = 2539)^43^ and prevention of progression of arterial disease and diabetes (POPADAD) (*n* = 1276)^44^ trials investigated aspirin in primary prevention but, being small and underpowered, failed to provide conclusive data. The recent ASCEND trial is the largest, longest and only adequately powered trial investigating primary prevention in DM, randomizing 15 480 DM patients without symptomatic cardiovascular disease to aspirin (100 mg daily) or placebo.^45^ Serious vascular events occurred in 8.5% of individuals on aspirin vs. 9.6% on placebo [RRR 12%, HR 0.88 (0.79–0.97); *P* = 0.01]. Major bleeding, according to the Bleeding Academic Research Consortium (BARC) 2, 3, and 5 categories,^46^ occurred in 4.1% and 3.2% in the aspirin and placebo arms, respectively, without significant differences in fatal or intracranial bleeding, although the absolute number of events was low (*Table 1*).^45^ Notably, >50% of major bleeding excess with aspirin was gastrointestinal. The number needed to treat (NNT)/number needed to harm (NNH) ratio was 0.8, favouring treatment. Importantly, BARC 2–5 bleeding criteria are less restrictive as compared to the Thrombolysis in Myocardial Infarction (TIMI) major criteria.^47–49^ Of note, there was no significant heterogeneity in the effect of aspirin according to the estimated vascular risk at baseline. A meta-analysis of 12 randomized controlled trials (RCTs) (34 227 individuals), including the ASCEND population, showed that aspirin reduces MACE by 11% compared with placebo [HR 0.89 (0.83–0.95)] (*Table 1*).^50^

**Table 1:** Primary prevention in diabetes

Study	Patients	Primary efficacy endpoint	Median follow-up	Predicted vs. observed incidence and expected benefit	Absolute and relative benefit	Absolute and relative harm	Comments
ATT meta-analysis (2009)^42^	95 000 patients from six primary prevention trials which included 4% of DM patients (*n* = 3818)	Stroke, MI, and CV death	NA	NA	*Overall population*: **Aspirin: 0.51%** **Control: 0.57%** **HR 0.88 (0.82–0.94)** *DM subgroup*: Aspirin: 1.63% Control: 1.87% HR 0.88 (0.67–1.15)	GI/extracranial bleed *Overall population*: **Aspirin: 0.10%** **Control: 0.07%** **HR 1.54 (1.30–1.82)** *DM subgroup*: Aspirin: 0.23%/year Control: 0.21%/year HR 1.10 (0.52–2.34)	NNT/NNH ratio: 0.83 No difference in fatal bleeding
JPAD (2008)^43^	2539 T2DM patients without a history of atherosclerotic disease	Sudden death; death from coronary, cerebrovascular, and aortic causes; non-fatal acute MI; UA; exertional angina; non-fatal ischaemic and haemorrhagic stroke; TIA; or non-fatal aortic and PVD	4.4 years	Predicted: 5.2%/year vs. Observed: 1.7%/year Expected benefit: 30% RRR	Aspirin: 5.4% Placebo: 6.7% HR 0.80 (0.58–1.10)	*Any GI bleeding*: Aspirin: *n* = 12 Placebo: *n* = 4	Observed primary endpoint rate ∼1/3 of predicted. Expected benefit likely unrealistic based on previous data (trial largely underpowered).
POPADAD (2008)^44^	1276 adults aged ≥40 years with T1DM or T2DM and ABI ≤0.99 (asymptomatic)	Death from CAD or stroke, non-fatal MI or stroke, or amputation for critical limb ischaemia; and death from CAD or stroke	6.7 years	Predicted: 28%/year vs. observed: 2.9%/year Expected benefit: 25% RRR	Aspirin: 18.2% Placebo: 18.3% HR 0.98 (0.76–1.26)	*Any GI bleeding*: Aspirin: 4.4% Placebo: 4.9% HR 0.90 (0.53–1.52)	Observed events were approx. 1/10 of predicted. The expected benefit was likely unrealistic based on previous data (trial was largely underpowered).
ASCEND (2018)^45^	**15 480** Patients aged ≥40 years with DM and no evident CV disease	Non-fatal MI, non-fatal stroke (excluding confirmed ICH), TIA, or death from any vascular cause (excluding confirmed ICH)	7.4 years	Predicted: 1.2–1.3%/year vs. Observed: 1.3%/year Expected benefit: 15% RRR	**Aspirin: 8.5%** **Placebo: 9.6%** **HR 0.88 (0.79–0.97)**	*BARC 2, 3, and 5 bleeding*: **Aspirin: 4.1%** **Placebo 3.2%** **HR 1.29 (1.09–1.52)** *ICH*: Aspirin: 0.7% Placebo: 0.6% HR 1.22 (0.82–1.81) *Fatal bleeding*: Aspirin: 0.2% Placebo: 0.2% HR 1.18 (0.61–2.30)	Consistency between predicted and observed incidence event rate NNT/NNH: 0.81
THEMIS (2019)^47^	19 220 patients with DM, ≥50 years, stable CAD with no previous MI or stroke Randomized to ticagrelor or placebo on a background of aspirin therapy	Stroke, MI, and CV death	3.3 years	Predicted benefit: 16% RRR Predicted: 2.5%/year vs. Observed: 2.5%/year	**Ticagrelor: 7.7%** **Placebo: 8.5%** **HR 0.90 (0.81–0.99)**	*TIMI major bleeding*: **Ticagrelor: 2.2%** **Placebo: 1.0%** **HR 2.32 (1.82–2.94)** *BARC 3–5 bleeding*: **Ticagrelor: 3.7%** **Placebo: 1.7%** **HR 2.36 (1.96–2.84)** *ICH*: **Ticagrelor: 0.7%** **Placebo: 0.5%** **HR 1.71 (1.18–2.48)**	High rate of ticagrelor discontinuation: Placebo 25% vs. Ticagrelor: 35% HR 1.50 (1.42–1.58) Predicted benefit higher than observed. NNT/NNH: 1.48 (TIMI-major defined bleeding)
Meta-analysis Seidu *et al*. (2019)^50^	34 227 participants with DM, individual patient data from 2306 participants	Stroke, MI, and CV death	5 years	NA	**Aspirin: 8.6%** **Control: 9.6%** **HR 0.89 (0.83–0.95)**	*Major bleeding*: Aspirin: 4% Control: 3.5% HR 1.30 (0.92–1.82)	

Summary of primary prevention studies.

Significant differences are reported in bold.

ABI, ankle-brachial index; BARC, Bleeding Academic Research Consortium; CAD, coronary artery disease; CV, cardiovascular; DM, diabetes mellitus; GI, gastrointestinal; HR, hazard ratio; ICH, intracranial haemorrhage; MI, myocardial infarction; NA, not applicable; NNH, number needed to harm; NNT, number needed to treat; PVD, peripheral vascular disease; RRR, relative risk reduction; T1DM, type 1 diabetes mellitus; T2DM, type 2 diabetes mellitus; TIA, transient ischaemic attack; TIMI, Thrombolysis in Myocardial Infarction; UA, unstable angina.

The THEMIS study tested intensification of antiplatelet regimen in 19 220 DM patients without previous myocardial infarction (MI) or stroke but with evidence of clinical CAD and already on low-dose aspirin therapy (*Table 1*).^47^ Individuals randomized to aspirin and ticagrelor had a modest reduction in vascular events compared with aspirin alone [7.7% and 8.5%, respectively, HR 0.90 (0.81–0.99); *P* = 0.04], associated with a 2.3-fold increase in TIMI major bleeding and a 1.7-fold increase in intracranial bleeding (*Table 1*), giving an unfavourable NNT/NNH ratio of 1.48 and arguing against routine DAPT with aspirin and ticagrelor in this population.

The recent European Society of Cardiology (ESC) guidelines indicate that those with DM and ≥1 organ damage or ≥3 major risk factors, or any risk factor and ≥10 years disease duration without organ damage, should be considered for primary prevention, in the absence of contraindications (Supplementary material online, *Table S2*) but routine use of aspirin for all DM individuals is not recommended.^51^ Overall, guidelines recommend aspirin monotherapy for DM patients with additional risk factor(s) and/or with an estimated annual risk of vascular events ≥1% (Supplementary material online, *Table S2*).^52–54^

## Secondary prevention in the absence of atrial tachyarrhythmias

### Following acute coronary events

ACS guidelines recommend DAPT comprising aspirin and prasugrel or ticagrelor, which applies to DM individuals.^3^ ^,^ ^51^ ^,^ ^55^ ^,^ ^56^ Patients with DM, however, exhibit enhanced platelet reactivity and reduced sensitivity to thienopyridines (but not ticagrelor),^57^ ^,^ ^58^ although the clinical significance of these biochemical observations remains unclear.

#### Aspirin

Guidelines recommend routine early administration of aspirin in ACS, which seems to offer similar benefits in individuals with and without DM. Based on pharmacodynamic studies in DM,^23^ ^,^ ^59^ ^,^ ^60^ the clinical benefit of twice-daily aspirin administration is being evaluated in the ANDAMAN trial (NCT02520921). Higher aspirin doses (300–325 mg vs. 75–100 mg) failed to reduce MACE 30 days post-ACS in the CURRENT-OASIS 7 study (23% had DM).^61^ The ongoing ADAPTABLE study is assessing alternative aspirin dosing, although this is not limited to the DM population.^62^ Moreover, ongoing studies are aiming to identify new low-dose aspirin formulations with potentially improved safety and efficacy profiles.^63^ ^,^ ^64^

#### P2Y_12_ inhibitors

In individuals with DM, prasugrel or ticagrelor combination with aspirin is preferred to clopidogrel, which shows reduced efficacy.^30^ ^,^ ^56^ ^,^ ^65–67^  *Post hoc* analysis of the DM population in the TRITON-TIMI 38 trial showed marked benefit of prasugrel over clopidogrel,^66^ while, in PLATO, the absolute benefit of ticagrelor over clopidogrel was greatest in patients with both DM and chronic kidney disease (CKD).^68^ In patients with ACS and insulin-requiring DM, ticagrelor may achieve more potent platelet inhibition than prasugrel,^57^ although the clinical significance is unclear. The recent ISAR-REACT 5 study, an open-label trial, demonstrated superiority of a prasugrel-based strategy over a ticagrelor-based strategy in reducing MACE in ACS patients.^69^ However, this was not the case in the DM subgroup: the composite primary endpoint (death, stroke, or MI) occurred in 11.2% and 13.0% in ticagrelor and prasugrel arms, respectively [HR 0.84 (0.58–1.24); *P* = 0.383] with treatment interaction shown for DM status (*P* = 0.0035).^70^ Bleeding complications were similar in ticagrelor- and prasugrel-treated DM individuals.

A difficulty with more potent oral P2Y_12_ inhibitors is the limited evidence in the older population who are at higher bleeding risk. Two smaller studies in ACS patients aged ≥70 years, of whom a third had DM, indicated that de-escalation from prasugrel or ticagrelor to clopidogrel may be safe.^71^ Moreover, platelet-function-guided de-escalation from prasugrel to clopidogrel at hospital discharge may be non-inferior to continued prasugrel but this strategy appears safer in those without DM.^72^ The TWILIGHT study (*n* = 7119) assessed safety of de-escalating DAPT, from ticagrelor plus aspirin to ticagrelor monotherapy, after 3 months of DAPT following high-risk percutaneous coronary intervention (PCI) for ACS or chronic coronary syndromes (CCS).^73^ Monotherapy reduced the primary endpoint of BARC type 2, 3, or 5 bleeding compared with DAPT at 12 months [4.0% vs. 7.1%, HR 0.56 (0.45–0.68); *P* < 0.001], similarly in those with and without DM, without increasing the secondary combined endpoint of death, MI, or stroke. More specifically, ticagrelor monotherapy in the DM subgroup did not increase ischaemic events compared with DAPT [4.6% vs. 5.9%; HR 0.77 (0.55–1.09); *P* = 0.14] but significantly decreased bleeding complications [4.5% vs. 6.7%; HR 0.65 (0.47–0.91); *P* = 0.012].^74^ The GLOBAL LEADERS trial randomized individuals undergoing PCI with drug-eluting stents for CCS or ACS to standard care (DAPT for 12 months followed by aspirin alone) or ticagrelor with aspirin for 1 month followed by ticagrelor monotherapy for 23 months. The study failed to show superiority for the intervention, although a trend towards a reduction in the primary composite endpoint of death or new Q-wave infarction was apparent [HR 0.87 (0.75–1.01); *P* = 0.073]. Risk of bleeding was almost identical in the two groups, regardless of DM status.^75^ ^,^ ^76^ The more recent analysis of the subgroup of DM individuals and CKD (higher risk of thrombosis and bleeding) showed no significant reduction in the primary endpoint with the intervention, although lower rates of the patient-oriented composite endpoint (POCE; death, stroke, site-reported MI/revascularization) were observed in the ticagrelor group compared with controls [20.6% vs. 25.9%, HR 0.74 (0.55–0.99)] with similar reduction in net adverse clinical events (POCE plus BARC 3 and 5 bleeding events) [22.7% vs. 28.3%, HR 0.75 (0.56–0.99)].^77^ Moreover, a recent meta-analysis has shown that, following PCI, monotherapy with a P2Y_12_ inhibitor is preferable to DAPT in older individuals and those with diabetes, CKD or multivessel disease due to reduction in bleeding risk.^78^ Therefore, de-escalation of DAPT may be an option in some patients, particularly when using potent P2Y_12_ inhibitors as monotherapy.^79^ However, de-escalation is perhaps best avoided in the DM population, given the high vascular risk, unless there are major concerns over bleeding risk and this remains an area for future research.

#### Glycoprotein IIb/IIIa inhibitors

GP inhibitor (GPI) use in ACS significantly reduced 30-day mortality, particularly in DM patients undergoing PCI, but this benefit was observed before routine P2Y_12_ inhibitor use.^80^ Abciximab reduced MACE in ACS patients undergoing PCI, even with clopidogrel pre-treatment, but the benefit in DM patients was less pronounced (abciximab has now been withdrawn in Europe).^81^ In contemporary practice using potent P2Y_12_ inhibitors, GPI therapy is mainly reserved for ‘bail-out’ in case of no-reflow or a thrombotic complication during PCI,^82^ although some benefit has been suggested also in opiate-treated patients undergoing emergency PCI.^83^

#### Very-low-dose non-VKA oral anticoagulant

**Figure 2:**
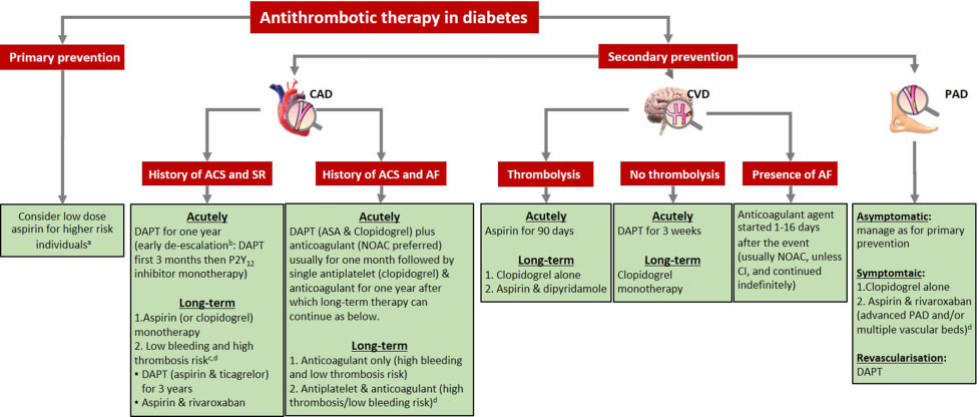
Summary of antiplatelet and anticoagulant therapies in individuals with diabetes and vascular disease. ACS: acute coronary syndrome; AF: atrial fibrillation; CAD: coronary artery disease; CVD: cerebrovascular disease; DAPT: dual antiplatelet therapy; NOAC: non-vitamin K antagonist oral anticoagulants; PAD: peripheral artery disease; SR: sinus rhythm. ^a^Diabetes mellitus individuals with ≥1 organ damage or ≥3 major risk factors, or any risk factor and ≥10-year disease duration without organ damage. ^b^Active bleeding or comorbidities with high bleeding risk. ^c^History of intracerebral haemorrhage or ischaemic stroke, history of other intracranial pathology, recent gastrointestinal bleeding or anaemia due to possible gastrointestinal blood loss, other gastrointestinal pathology associated with increased bleeding risk, liver failure, bleeding diathesis or coagulopathy, extreme old age or frailty, or renal failure requiring dialysis or with estimated glomerular filtration rate <15 mL/min/1.73 m^2^. ^d^Diffuse multivessel CAD with at least one of the following: diabetes mellitus requiring medication, recurrent myocardial infarction, PAD, or chronic kidney disease with estimated glomerular filtration rate 15–59 mL/min/1.73 m^2^.

In the ATLAS ACS 2-TIMI 51 trial, the addition of very-low-dose rivaroxaban (2.5 mg twice daily) to DAPT (in the form of aspirin and clopidogrel) in ACS patients, of whom 32% had DM, significantly reduced MACE compared to placebo but the DM group appeared to derive less benefit.^84^ An increase in bleeding events, including intracranial, was documented and therefore this triple therapy (TT) can only be advocated for individuals at very high vascular risk with relatively low bleeding risk, accepting the challenge that ischaemic and bleeding risk factors overlap substantially.^55^ A summary of the agents used following ACS is given in *Figure 2*.

### Long-term therapy for secondary prevention

Default practice is DAPT for 1 year post-ACS followed by antiplatelet monotherapy, usually with aspirin. However, DM patients have increased long-term risk of recurrent atherothrombotic events and should be carefully considered for more intensive long-term antithrombotic therapy.^85^

The CAPRIE trial (*n* = 19 185) showed that clopidogrel for secondary prevention (75 mg daily) reduced the composite endpoint of ischaemic stroke, MI, or vascular death compared with daily 325 mg aspirin [5.32% vs. 5.83% (0.3–16.5); *P* = 0.043], mostly driven by PAD events, which showed a significant heterogeneity vs. MI and stroke.^86^ DM patients (*n* = 3866) showed a similar pattern (*P* = 0.36 for interaction) but with an amplified absolute risk reduction (15.6% vs. 17.7%; *P* = 0.042), without an increase in bleeding events.^87^ Given the high aspirin dose used, it is difficult to recommend a routine switch to clopidogrel for secondary prevention, except in special clinical scenarios, such as individuals with PAD (discussed below). The benefit of 12 vs. 30 months of DAPT, mostly consisting of aspirin and clopidogrel, was tested in the DAPT trial,^88^ including 9961 individuals who had not experienced ischaemic or bleeding events at 1 year post-PCI. Prolonged DAPT significantly reduced the composite of all-cause mortality, MI, or stroke [4.3% vs. 5.9%, HR 0.71 (0.59–0.85); *P* < 0.001] but at the expense of increased moderate/severe bleeding events [2.5% vs. 1.6%, HR 1.61 (1.21–2.16); *P* = 0.001]. However, in DM patients (*n* = 3391), this strategy did not affect the composite outcome [6.6% vs. 7.0%, HR 0.92 (0.71–1.20); *P* = 0.55] although the limitation of subgroup analysis should be acknowledged.^89^ ^,^ ^90^

The PEGASUS-TIMI 54 trial evaluated prolonged ticagrelor use in 21 162 patients with a history of MI 1–3 years prior to enrolment. Patients also needed to have at least one additional risk factor, which included DM requiring medication.^91^ Patients were randomized to either one of two doses of ticagrelor (90 mg twice daily or 60 mg twice daily) or placebo in addition to aspirin. At 3 years, the primary efficacy endpoint (cardiovascular death, MI, and stroke) was reduced with ticagrelor 60 mg twice daily compared with placebo [7.8% vs. 9.0% in placebo, HR 0.84 (0.74–0.95); *P* = 0.004]. TIMI major bleeding events increased [2.30% vs. 1.06% in placebo, HR 2.32 (1.68–3.21); *P* < 0.001], but no difference was detected in fatal or intracranial bleeding. Similar data were documented in the DM subgroup (*n* = 6806, 32% of study population) with a higher absolute risk reduction in the 60 mg twice daily ticagrelor arm compared with placebo (11.6% vs. 10.0% in DM subgroup and 7.8% vs. 6.7% in non-DM subgroup). TIMI major bleeding in those with DM was higher in ticagrelor-treated individuals compared with placebo [2.5% and 1.0%, HR 2.5 (1.4–4.4); *P* = 0.0004], an increase that was similar to the non-DM group (2.39%; *P* = 0.89).^91^

The COMPASS study in patients with either prior MI or multivessel CAD (*n* = 27 395; 38% with DM) showed that rivaroxaban 2.5 mg twice daily added to low-dose aspirin reduced the risk of MACE compared with aspirin alone [4.1% vs. 5.4%, HR 0.76 (0.66–0.86); *P* < 0.001],^92^ making this an option,^85^ ^,^ ^92^ ^,^ ^93^ particularly in DM patients who showed greater absolute net benefit.^94^ While the combination therapy increased bleeding risk, there was still a net clinical benefit. Vorapaxar was investigated in the TRA 2°P-TIMI 50 study, detailed in Supplementary material online given the limited clinical use of this agent.^95–97^

In summary, following ACS, DAPT for 1 year is the current standard of care. De-escalation of DAPT intensity or duration may be considered after 3 months if bleeding concerns prevail.^98^ One year post-ACS, options include switching to aspirin monotherapy or, in high ischaemic and low bleeding risk DM patients, continuation of dual antithrombotic therapy in the form of aspirin and low-dose ticagrelor (PEGASUS-TIMI 54 study) or aspirin and very-low-dose rivaroxaban (COMPASS trial).^3^ ^,^ ^51^ ^,^ ^85^ Studies addressing antithrombotic agents for secondary prevention are summarized in *Table 2* and Supplementary material online, Figure *S*1.

**Table 2:** Secondary prevention in diabetes

	Sample size	Population	Intervention	Control	Diabetes (%)	Primary endpoint	Duration of follow-up	Relative and absolute benefit	Relative and absolute harm	Comments
CAPRIE (1996)^86^ RCT 1:1	19 185	Prior ischaemic stroke (within 1 week–6 months), recent MI (within 35 days), or symptomatic atherosclerotic PAD	Clopidogrel (75 mg)	Aspirin (325 mg)	20	Aggregate of MI, ischaemic stroke, and vascular death	1–3 years	**5.32% vs. 5.83% RRR 8.7%** **(0.30–16.5%); *P* = 0.043**	**GI bleed 1.99% vs. 2.66%; *P* < 0.002**	Treatment effect by subgroup suggests heterogeneity in response with a benefit in PAD, but not is post-MI or stroke patients
DAPT (2014)^88^ RCT 1:1	9961	Prior coronary stent with DES after 12 months of DAPT (thienopyridine and aspirin)	Thienopyridine (clopidogrel 65% or prasugrel 35%)	Placebo	30	(i) Stent thrombosis and (ii) MACCE (death, MI, or stroke)	18 months	**(i) Stent thrombosis 0.4% vs. 1.4%; HR 0.29 (0.17–0.48); *P* < 0.001** **(ii) MACCE 4.3% vs. 5.9% HR 0.71 (0.59–0.85); *P* < 0.001**	**Moderate–severe bleeding 2.5% vs. 1.6%; *P* = 0.001**	
PEGASUS-TIMI 54 (2015)^91^ RCT 1:1:1	21 162	MI 1–3 years earlier	Ticagrelor 90 mg b.i.d.	vs. placebo	32	Composite of CV death, MI, or stroke	33 months	**7.85% vs. 9.04% HR 0.85 (0.75–0.96); *P* = 0.008**	**TIMI major bleeding** **2.60% vs. 1.06%, *P* < 0.001**	No difference was detected in fatal or intracranial bleeding
			Ticagrelor 60 mg b.i.d.					**7.77% vs. 9.04% HR 0.84 (0.74–0.95); *P* = 0.004**	**TIMI major bleeding** **2.30% vs. 1.06%;** ***P* < 0.001**	
TRA 2P-TIMI 50 (2012)^95^ RCT 1:1	26 449	History of MI, ischaemic stroke, or PAD	Vorapaxar (2.5 mg daily)	Placebo	25	Composite of death from CV causes, MI, or stroke	30 months	**9.3% vs. 10.5% HR 0.87 (0.80–0.94); *P* < 0.001**	**Moderate or severe bleeding** **4.2% vs. 2.5%,** **HR 1.66 (1.43–1.93); *P* < 0.001** **ICH** **1.0% vs. 0.5%; *P* < 0.001**	Premature trial termination at 2 years, due to safety concerns over ICH in patients with history of stroke
COMPASS (2017)^92^ RCT 1:1:1	27 395	Stable CAD, PAD, or both	Rivaroxaban (2.5 mg b.i.d.) plus aspirin (100 mg/day)	Aspirin (100 mg/day)	38	Composite of CV death, stroke, or MI	23 months	**4.1% vs. 5.4% HR 0.76 (0.66–0.86); *P* < 0.001**	**Any bleeding** **3.1% vs. 1.9%** **HR 1.70 (1.40–2.05); *P* < 0.001**	Major bleeding was not significantly different
			Rivaroxaban (5 mg b.i.d.)					4.9% vs. 5.4% HR 0.90 (0.79–1.03); *P* = 0.12	**Any bleeding** **2.8% vs. 1.9%** **HR 1.51 (1.25–1.84); *P* < 0.001**	

Long-term therapy for secondary prevention trials in patients with established cardiovascular disease.

This is a general guide and healthcare professionals should follow local guidelines as appropriate.

Significant differences are highlighted in bold.

b.i.d., twice daily; CAD, coronary artery disease; CV, cardiovascular; DAPT, dual antiplatelet therapy; DES, drug-eluting stent; GI, gastrointestinal; HR, hazard ratio; ICH, intracranial haemorrhage; MACE, major adverse cardiovascular or cerebrovascular events; MI, myocardial infarction; PAD, peripheral arterial disease; RCT, randomized controlled trial; RRR, relative risk reduction.

### Antithrombotic therapy in the presence of atrial fibrillation

Individuals with DM have a 40% greater risk of developing atrial fibrillation (AF) compared to those without DM.^99^ ^,^ ^100^ Whilst epidemiological data suggest a causal association, the effect of confounders cannot be excluded (further discussed in Supplementary material online, *S1*).^101–109^

#### Antithrombotic therapy

Oral anticoagulation (OAC) is recommended for male and female AF patients with CHA_2_DS_2_-VASc score ≥2 and ≥3, respectively, but can also be considered in those with lower scores on an individual basis.^51^ In those without absolute indication for VKAs (e.g. mechanical valve or moderate/severe mitral stenosis), NOACs are preferred due to lower rates of severe bleeding and reduced monitoring requirements.^51^ ^,^ ^110^ In studies comparing NOACs with VKA in non-valvular AF, about a third of individuals had DM and showed similar RRR to those without DM.^111^ However, given the increased risk in DM, therapy with NOACs translated into a greater absolute benefit, while rates of major bleeding are similar regardless of DM status. If OAC is contraindicated, percutaneous left atrial appendage closure is an option in those with or without DM.^112^

##### Antithrombotic therapy in patients with atrial fibrillation and acute coronary syndrome

Patients with AF on OAC who develop ACS and/or undergo PCI generally require combination treatment with OAC and antiplatelet therapy, with NOACs preferred over VKA.^82^ ^,^ ^85^ ^,^ ^113^ For TT with OAC and DAPT, clopidogrel is recommended over more potent P2Y_12_ inhibitors.^82^ ^,^ ^85^ ^,^ ^113^

After elective PCI in patients with AF who require OAC, a switch from TT (DAPT and OAC) to dual therapy (OAC and single antiplatelet therapy) should be considered in those at low risk of stent thrombosis or when bleeding risk is high. North American guidance encourages dual therapy with early aspirin cessation (i.e. by the time of hospital discharge).^114^ ^,^ ^115^ ESC guidance endorses TT for at least 1 month if stent thrombosis risk outweighs bleeding risk, followed by an antiplatelet agent (usually clopidogrel) and OAC until 12 months post-PCI, and thereafter OAC monotherapy, unless long-term dual therapy is considered due to very high ischaemic risk with low bleeding risk.^82^ ^,^ ^85^ ^,^ ^102^ ^,^ ^113^ Similar differences in international guidance exist for AF patients with ACS treated with stent implantation.^55^ ^,^ ^56^ ^,^ ^116^ ^,^ ^117^ The above applies to all individuals regardless of DM status as no specific studies have been conducted in this group. A summary of the agents used in patients with AF and ACS is provided in *Figure 2*.

## Peripheral artery disease

PAD is thought to affect 202 million people worldwide^118^ and these individuals are at a higher MACE risk,^119^ with an even higher event rate with two or more arterial beds affected.^119^ ^,^ ^120^

### Asymptomatic peripheral artery disease

Two relatively small trials have compared aspirin vs. placebo in asymptomatic PAD, the POPADAD trial^44^ specific to DM and the AAA trial,^121^ which included 3% (*n* = 88) of DM patients. Neither showed a difference in MACE in a combined overall cohort of 4626 patients (1256 with DM). While it can be argued these studies were underpowered, guidelines generally do not support routine antiplatelet therapy for asymptomatic lower extremity arterial disease and these individuals are best managed as per primary prevention recommendations described above.

### Symptomatic peripheral artery disease

A meta-analysis of 17 000 individuals with symptomatic PAD has shown that aspirin reduced serious vascular events by 18.2% per year (*P* < 0.0001), marginally offset by a non-significant increase in haemorrhagic stroke.^42^

Subgroup analysis of 6452 patients with PAD (21% with DM) in the CAPRIE study showed potentially greater benefit with clopidogrel vs. aspirin [3.71% vs. 4.86%; RRR 24% (8.9–36.2); *P* = 0.0028] compared with the overall study population [5.32% vs. 5.83%; RRR 8.7% (0.3–16.5); *P* = 0.043].^86^ The EUCLID trial compared ticagrelor and clopidogrel in 13 885 patients with symptomatic PAD (38.4% with DM), finding no difference in MACE [10.8% vs. 10.6%, HR 1.02 (0.92–1.13); *P* = 0.65] or major bleeding over a median follow-up of 30 months, regardless of DM status.^122^

A subgroup analysis of PAD patients in the CHARISMA trial (*n* = 3096, 36.2% with DM) showed no significant difference in MACE comparing aspirin and clopidogrel therapy with aspirin and placebo [7.6% vs. 8.9%, HR 0.85 (0.66–1.08); *P* = 0.18], similar to the overall trial cohort [6.8% vs. 7.3%, HR 0.93 (0.83–1.05); *P* = 0.22].^123^ There was an increase in minor bleeding with DAPT [34.4% vs. 20.8%, odds ratio 1.99 (1.69–2.34); *P* < 0.001] but no difference in severe/moderate bleeding. The role of vorapaxar in PAD is described in the Supplementary material online.^124^

A systematic review and network meta-analysis reported that aspirin, ticlopidine, and ticagrelor or clopidogrel used as monotherapy (or in combination with aspirin) were effective in reducing MACE in patients with PAD, and that ticlopidine, vorapaxar, and DAPT increased bleeding risk.^125^ Clopidogrel had the best balance between efficacy [MACE RRR 0.72 (0.58–0.91); NNT 80] and safety profile, making it the preferred agent to use.

There were 7470 patients with PAD in the COMPASS trial, 44% of whom had DM with a median follow-up of 21 months.^126^ The combination of rivaroxaban and aspirin reduced MACE and major adverse limb events vs. aspirin alone to a similar degree in those with DM [8% vs. 12%, HR 0.69 (0.53–0.91)] or without [5% vs. 7%, HR 0.69 (0.50–0.94)]. This was associated with a lower incidence of major adverse limb events in the whole group [1% vs. 2%, HR 0.54 (0.35–0.84); *P* = 0.005], including lower incidence of major amputation [HR 0.30 (0.11–0.80); *P* = 0.011].

### Post-revascularization

Limited evidence suggests that DAPT with aspirin and clopidogrel is beneficial after lower limb revascularization, particularly following prosthetic bypass.^125^ ^,^ ^127^ Similarly, warfarin, with or without aspirin, may improve graft patency after vein bypass.^128^ ^,^ ^129^ The VOYAGER PAD trial, assessing aspirin plus rivaroxaban vs. aspirin post-lower limb revascularization, included 6564 individuals (40% had DM) and combination therapy reduced the composite of acute limb ischaemia, major amputation, MI, ischaemic stroke, or cardiovascular death [HR 0.85 (0.76–0.96); *P* = 0.009]. A trend towards an increase in TIMI major bleeding, but not BARC bleeding, was documented with the combination therapy [HR 1.43 (0.97–2.10); *P* = 0.07].^130^ Results of the DM subgroup analysis are awaited. Antithrombotic management of individuals with DM and PAD is summarized in *Table 3* and Supplementary material online, *Figure S1*.

**Table 3:** Antithrombotic therapy in peripheral vascular disease

Trial	Sample size	Population	Investigation	Control	% with diabetes	Follow-up	Outcomes	Absolute and relative benefits	Absolute and relative harms	Other comments
Asymptomatic										
POPADAD (2008)^44^	1276	Type 1 or 2 diabetes mellitus and ABPI ≤0.99 with no PAD symptoms	Aspirin 100 mg ± antioxidant	Placebo ± antioxidant	100%	Median 6.7 years	MACCE or above ankle amputation for critical limb ischaemia	18.2% vs. 18.3% HR 0.98 (0.76–1.26); *P* = 0.86	GI bleed 4.4% vs. 4.9% HR 0.90 (0.53–1.52); *P* = 0.69	
AAA (2010)^121^	3350	ABPI ≤0.95; free from clinical CV disease	Aspirin 100 mg	Placebo	3%	Mean 8.2 years	MACCE or revascularization	13.7 vs. 13.3 events/1000 person-years HR 1.03 (0.84–1.27)	Major bleed 2.0% vs. 1.2% HR 1.71 (0.99–2.97)	
Symptomatic										
ATT Collaboration (2009)^42^	17 000	Meta-analysis of secondary prevention trials (not PAD specific)	Aspirin 75–500 mg	No aspirin	Not stated	NA	MACCE	**6.7% vs. 8.2% per year;** **HR 0.81 (0.75–0.87); *P* < 0.0001**	Major extracranial bleed (incompletely reported) 23 vs. 6 events HR 2.69 (1.25–5.76)	Non-significant increase haemorrhagic stroke, significant decrease ischaemic stroke and coronary events
CAPRIE (1996)^86^	6452	Symptomatic PAD and ABPI ≤0.85; or symptomatic PAD with previous amputation or revascularization	Clopidogrel 75 mg	Aspirin 325 mg	21%	Mean 1.9 years	MACCE	**3.71% vs. 4.86% per year;** **RR 23.8% (8.9–36.2); *P* = 0.0028**	**GI bleed 1.99% vs. 2.66%; *P* < 0.002** (for the whole group)	No difference in amputation rate across CAPRIE cohorts; not reported specific to PAD subgroup
EUCLID (2017)^122^	13 885	PAD with ABPI ≤0.8 or previous lower limb revascularization >30 days before randomization	Ticagrelor 90 mg b.i.d.	Clopidogrel 75 mg	38%	Median 30 months	MACCE	10.8% vs. 10.6% HR 1.02 (0.92–1.13); *P* = 0.65	TIMI major bleeding 1.6% vs. 1.6% HR 1.10 (0.84–1.43); *P* = 0.49	
							Lower limb revascularization	12.2% vs. 12.8% HR 0.95 (0.87–1.05); *P* = 0.30		
CHARISMA subgroup PAD (2009)^123^	3096 (2838 symptomatic, 258 asymptomatic)	Symptomatic PAD and ABPI ≤0.85; or symptomatic PAD with previous amputation or revascularization; asymptomatic with APBI <0.9 identified within those with other eligibility for CHARISMA study	Aspirin 75–162 mg + clopidogrel 75 mg (DAPT)	Aspirin 75–162 mg + placebo	36%	Median 28 months	MACCE	7.6% vs. 8.9% HR 0.85 (0.66–1.08); *P* = 0.183	Severe bleeding 1.7% vs. 1.7% HR 0.97 (0.56–1.66); *P* = 0.90 Minor bleeding 34.4% vs. 20.8% HR 1.99 (1.69–2.34); *P* < 0.001	Non-significant trend towards increase of fatal, intracranial, and moderate bleeding with DAPT
TRA2°P-TIMI 50 (2013)^124^	3787	Symptomatic PAD and ABPI <0.85 or previous lower limb revascularization	Vorapaxar 2.5 mg	Placebo	36%	Median 36 months	MACCE	11.3% vs. 11.9% HR 0.94 (0.78–1.14); *P* = 0.53	GUSTO moderate/severe bleeding: **7.4% vs. 4.5%** **HR 1.62 (1.21–2.18); *P* = 0.001**	
							Acute limb ischaemia	**2.3% vs. 3.9%** **HR 0.58 (0.39–0.86); *P* = 0.006**		
							Revascularization	**18.4% vs. 22.2%** **HR 0.84 (0.73–0.97); *P* = 0.017**		
COMPASS (2018)^126^	7470 (4129 symptomatic lower limb; 1422 asymptomatic lower limb; 1919 carotid disease)	Previous lower limb revascularization or amputation; symptomatic PAD and ABPI <0.9 or stenosis ≥50% on arterial imaging; carotid revascularization or asymptomatic carotid artery stenosis ≥50%	Rivaroxaban 2.5 mg b.i.d + aspirin 100 mg	Aspirin 100 mg + placebo	44%	Median 21 months	MACCE	**5% vs. 7%** **HR 0.72 (0.57–0.90); *P* = 0.005**	**Major bleeding 3.1% vs. 1.9%** **HR 1.61 (1.12–2.31); *P* = 0.009**	
							Major adverse limb event (acute/chronic ischaemia; amputation)	**1.2% vs. 2.2%** **HR 0.54 (0.35–0.84); *P* = 0.005**		
Post-revascularization										
CASPAR (2010)^127^	851	Vascular bypass graft for treatment of PAD	Aspirin 75–100 mg + clopidogrel 75 mg (DAPT)	Aspirin 75–100 mg + placebo	38%	Median 12 months	Graft occlusion/revascularization/amputation/death	All grafts 35.4% vs. 35.0% HR 0.98 (0.78–1.23) Venous 23.8% vs. 20.0% HR 1.25 (0.94–1.67) **Prosthetic** **37.5% vs. 52.8%** **HR 0.65 (0.45–0.95**)**; *P* = 0.025**	**Total bleeding** **16.7% vs. 7.1%; *P* < 0.001** Severe bleeding 2.1% vs. 1.2%; *P* = NS	Graft occlusion HR 0.63 (0.42–0.93) and amputation HR 0.48 (0.24–0.96) **significantly reduced in prosthetic** but not vein bypass
BOA (2000)^128^	2690	Infrainguinal bypass graft for obstructive arterial disease	Oral anticoagulants (target INR 3.0–4.5)	Pulverized carbasalate calcium 100 mg (equivalent to aspirin 80 mg)	26%	Mean 21 months	Occlusion	23.2% vs. 24.3% HR 0.95 (0.82–1.11)	Total bleeding 119 vs. 59 events Fatal bleeding 16 vs. 12 events Gastrointestinal bleeding 51 vs. 29 events Intracranial bleeding 18 vs. 4 events	Fatal intracranial bleeding events were higher (8 vs. 3 events) in oral anticoagulants, whereas bleeding events in other sites were similar between groups
							MACE plus amputation	18.7% vs. 20.8% HR 0.89 (0.75–1.06)		
							Vein graft occlusion	**14.3% vs. 20.3%** **HR 0.69 (0.54–0.88)**		
							Non-vein grafts occlusion	**36% vs. 30%** **HR 1.26 (1.03–1.55)**		
Sarac *et al*. (1998)^129^	56	Infrainguinal bypass with autogenous vein and deemed high risk for graft occlusion (suboptimal venous conduit, poor arterial runoff or redo bypass)	Warfarin (target INR 2–3) + aspirin 325 mg	Aspirin 325 mg	64%	Not stated (outcomes derived from Kaplan–Meier survival curves)	30-day graft patency	97.3% vs. 85.2%; *P* = 0.07	**Haematoma** **32% vs. 4%; *P* = 0.004** GI bleeding 3% vs. 11%; *P* = NS Intracranial bleeding 3% vs. 4%; *P* = NS	
							30-day amputation rate	**0% vs. 11.1%; *P* = 0.04**		
							3-year primary assisted patency:	77% vs. 56%; *P* = 0.05		
							3-year secondary patency	**81% vs. 56%; *P* = 0.02**		
VOYAGER-PAD (2020)^130^	6564	Post-lower limb revascularization	Rivaroxaban 2.5 mg b.i.d. + aspirin 100 mg	Aspirin 100 mg + placebo	40%	Median 28 months	MACE plus acute limb ischaemia or amputation	**15.5% vs. 17.8%** **HR 0.85 (0.76–0.96)**	Major bleeding 1.9% vs. 1.35% HR 1.43 (0.97–2.10); *P* = 0.07	

Summary of antiplatelet and anticoagulant studies in individuals with peripheral vascular disease.

Significant differences are highlighted in bold.

ABPI, ankle brachial pressure index; b.i.d., twice daily; CV, cardiovascular; DAPT, dual antiplatelet therapy; GI, gastrointestinal; HR, hazard ratio; INR, international normalized ratio; MACE, major adverse cardiovascular or cerebrovascular events; NA, not available; PAD, peripheral artery disease.

## Treatment of individuals with cerebrovascular disease

Due to lack of DM-specific studies, antithrombotic therapy in individuals sustaining a stroke is similar regardless of DM status and therefore studies are discussed accordingly.

### Following acute events

In acute severe ischaemic stroke, reperfusion is attempted either through thrombolysis or endovascular thrombectomy, followed by antiplatelet monotherapy, usually with aspirin, administered 24 h later.^131^ ^,^ ^132^ Ticagrelor monotherapy showed no superiority over aspirin^133^ and, therefore, is only recommended if aspirin is contraindicated.^132^

In those with minor events [National Institute of Health Stroke Scale (NIHSS) score ≤3], high-risk transient ischaemic attack (TIA) (ABCD2 score ≥4) or TIA not requiring thrombolysis or invasive measures, antiplatelet therapy can be immediately started provided haemorrhagic stroke is excluded. DAPT (aspirin and clopidogrel) is recommended given findings from the CHANCE and POINT trials (21% and 28% of the study population had diabetes, respectively),^134^ ^,^ ^135^ starting within 24 h of the event for 21 days followed by clopidogrel only.^132^ While severe haemorrhagic events showed no increase in CHANCE, a doubling was noticed in POINT (*Table 4* and Supplementary material online, *Figure S1*), although the benefit of DAPT still outweighed bleeding risk.^135^ The more recent THALES trial randomized 11 016 individuals (29% with diabetes), with ischaemic stroke or TIA (NIHSS score ≤5; 29% with DM) within 24 h of presentation to DAPT with ticagrelor and aspirin or aspirin alone for 30 days. The primary composite outcome of stroke or death at 30 days occurred in 5.5% in the combination group vs. 6.6% in those on aspirin alone [HR 0.83 (0.71–0.96); *P* = 0.02] but incidence of disability showed no difference, while DAPT was associated with increased rate of severe bleeding (0.5% vs. 0.1%; *P* = 0.001).^136^ There was no suggestion in any of the studies that the diabetes subgroup behaved differently and therefore DAPT in DM should be initiated within 24 h following acute minor stroke not requiring thrombolysis or thrombectomy and continued for 21–30 days. Future work is required to clarify the optimal duration of DAPT after acute minor stroke, or the benefit of DAPT following reperfusion therapy.^132^ ^,^ ^137^

**Table 4:** Antithrombotic therapy in cerebrovascular disease

Antiplatelet randomized trial for secondary prevention in patients with acute minor ischaemic stroke/high-risk TIA								
Study	Patients	Intervention	Control	Follow-up	Composite vascular events (stroke, MI or CVD death)	Recurrent ischaemic stroke	Intracranial haemorrhage	Major haemorrhage
CHANCE (2013)^134^ (*N* = 5170; 21.1% with DM)	Minor stroke (NIHSS <3)/high-risk TIA, onset <24 h	Clopidogrel 300 mg loading then 75 mg/day on days 2–90 Aspirin 75–300 mg/day on days 2–21	Clopidogrel 75 mg/day on days 1–90	90 days	**8.4% vs. 11.9%** **HR 0.69 (0.58–0.82)**	**7.9% vs. 11.4%** **HR 0.67 (0.56–0.81)**	0.3% vs. 0.3% HR 1.01 (0.38–2.70) *(haemorrhagic stroke)*	0.2% vs. 0.2% HR 0.94 (0.24–3.79) *(severe bleeding)* 0.1% vs. 0.2% HR 0.73 (0.16–3.26) *(moderate bleeding)*
POINT (2018)^135^ (*N* = 4881; 27.5% with DM)	Minor stroke (NIHSS <3)/high-risk TIA, onset <12 h	Clopidogrel 600 mg loading then 75 mg/day on days 2–90 Aspirin 50–325 mg/day on days 2–21	Aspirin 50–325 mg on days 1–90 (recommend 162 mg/day on day 1–5, then 81 mg/day afterward)	90 days	**5.0% vs. 6.5%** **HR 0.75 (0.59–0.95)**	**4.6% vs. 6.3%** **HR 0.72 (0.56–0.92)**	0.2% vs. 0.1% HR 1.68 (0.40–7.03) *(haemorrhagic stroke)*	**0.9% vs. 0.4%** **HR 2.32 (1.10–4.87)**
SOCRATES (2016)^133^ (*N* = 6589; 24.3% with DM)	Non-severe stroke (NIHSS <5)/high-risk TIA, onset <24 h	Ticagrelor 180 mg loading then 90 mg b.i.d. on days 2–90	Aspirin 300 mg loading then 100 mg/day on days 2–90	90 days	6.5% vs. 7.2% HR 0.89 (0.78–1.01)	5.9% vs. 6.6% HR 0.87 (0.76–1.00)	0.2% vs. 0.3% HR 0.68 (0.33–1.41)	0.5% vs. 0.6% HR 0.83 (0.52–1.34)
THALES (2020)^136^ (*N* = 11 016; 28.6% with DM)	Mild–moderate stroke (NIHSS <5)/high-risk TIA (ABCD2 > 6), onset <24 h	Ticagrelor 180 mg loading then 90 mg b.i.d. on days 2–30 Aspirin 300–325 mg loading then 75 to 100 mg/day on days 2–30	Aspirin 300–325 mg loading then 75–100 mg o.d. on days 2–30	30 days	**5.5% vs. 6.6%** **HR 0.83 (0.71–0.96)** ***(stroke or death)***	**5.0% vs. 6.3%** **HR 0.79 (0.68–0.93)**	**0.4% vs. 0.1%** **HR 3.33 (1.34–8.28)**	**0.5% vs. 0.1%** **HR 3.99 (1.74–9.14)**

Anti-platelet randomized trials for long-term secondary prevention in patients with previous non-cardioembolic ischaemic stroke/TIA								
Study	Patient	Intervention	Control	Follow-up	Non-fatal MI, non-fatal stroke, death from vascular causes	Recurrent ischaemic stroke	Intracranial haemorrhage	Major haemorrhage
CAPRIE (1996)^86^ (*N* = 19 185; 20% with DM)	Recent MI, PAD, or stroke	Clopidogrel 75 mg/day	Aspirin 325 mg/day	1.9 years	**5.32% vs. 5.83%** **HR 0.91 (0.84–1.00)**	Not reported	0.35% vs. 0.49%	1.38% vs. 1.55% *(severe bleeding)*
CAPRIE (1996)^86^ stroke subgroup (*N* = 6431; 25% with DM)	Recent stroke	Clopidogrel 75 mg/day	Aspirin 325 mg/day		7.15% vs. 7.71% HR 0.91 (0.81–1.06)	5.2% vs. 5.7%	Not reported	Not reported
ESPRIT (2006)^140^ (*N* = 2739; 18.7% with DM)	Recent minor stroke/TIA (mRS <3) within 6 months	Dipyridamole 200 mg b.i.d. *plus* Aspirin 30–325 mg/day (median 75 mg/day)	Aspirin 30–325 mg/day (median 75 mg/day)	3.5 years	**3.3% vs. 4.3%** **HR 0.78 (0·63–0·97)**	2.1% vs. 2.6% HR 0.84 (0.64–1.10)	0.8% vs. 1.5%	2.5% vs. 3.9%
PRoFESS (2008)^141^ (*N* = 20 332; 28% with DM)	Recent stroke within 90 days	Extended release dipyridamole 200 mg b.i.d. *plus* Aspirin 25 mg b.i.d.	Clopidogrel 75 mg/day	2.5 year	13.1% vs. 13.1% HR 0.99 (0.92–1.07)	7.7% vs. 7.9% HR 0.97 (0.88–1.07)	**1.4% vs. 1.0%** **HR 1.42 (1.11–1.83)**	**4.1% vs. 3.6%** **HR 1.15 (1.00–1.32)**
MATCH (2004)^145^ (*N* = 7599; 68% with DM)	Recent stroke/TIA within 3 months plus >1 risk factors	Clopidogrel 75 mg/day *plus* Aspirin 75 mg/day	Clopidogrel 75 mg/day	1.5 years	16% vs. 17% HR 0.94 (0.84–1.05)	8% vs. 9% HR 0.93 (0.80–1.09)	1.1% vs. 0.7%	**1.9% vs. 0.6%** **(*P* < 0.001)**
CHARISMA (2006)^142^ (*N* = 15 603; 42.7% with DM)	Atherosclerotic risks, CVD, stroke/TIA within 5 years	Clopidogrel 75 mg/day *plus* Aspirin 75–162 mg/day	Aspirin 75–162 mg/day	2.3 years	6.8% vs. 7.3% HR 0.93 (0.83–1.05)	1.7% vs. 2.1% HR 0.81 (0.64–1.02)	0.3% vs. 0.3% HR 0.96 (0.56–1.65)	**2.1% vs. 1.3%** **HR 1.62 (1.27–2.08)**
SPS3 (2012)^143^ (*N* = 3020; 36.5% with DM)	Symptomatic lacuna stroke within 6 months	Clopidogrel 75 mg/day *plus* Aspirin 325 mg/day	Aspirin 325 mg/day	3.4 years	3.1% vs. 3.4% HR 0.89 (0.72–1.11)	2.0% vs. 2.4% HR 0.82 (0.63–1.09)	0.4% vs. 0.3% HR 1.65 (0.83–3.31)	**2.1% vs. 1.1%** **HR 1.97 (1.41–2.71)**
Greving *et al*. (2019)^144^ (*N* = 43 112; 33.3% with DM)	Meta-analysis (6 RCTs)	Clopidogrel	Aspirin	2.0 years	**0.88 (0.78–0.98)**	0.91 (0.81–1.02)	**0.63 (0.43–0.91)**	**0.76 (0.63–0.91)**
		Aspirin/dipyridamole			**0.83 (0.74–0.94)**	**0.86 (0.76–0.97)**	0.88 (0.60–1.31)	0.86 (0.71–1.05)
		Aspirin/clopidogrel			**0.83 (0.71–0.96)**	**0.83 (0.71–0.97)**	1.19 (0.68–2.08)	**1.63 (1.29–2.07)**
		Aspirin/dipyridamole	Clopidogrel		0.95 (0.85–1.06)	0.95 (0.87–1.04)	**1.40 (1.08–1.82)**	**1.14 (1.00–1.30)**
		Aspirin/clopidogrel			0.94 (0.82–1.08)	0.91 (0.80–1.04)	**1.88 (1.12–3.16)**	**2.16 (1.72–2.71)**
		Aspirin/clopidogrel	Aspirin/dipyridamole		0.99 (0.84–1.17)	0.96 (0.82–1.13)	1.34 (0.77–2.36)	**1.89 (1.47–2.42)**

Anti-coagulant randomized trials for primary and secondary preventions in patients with atrial fibrillation								
Study	Patients	Intervention	Control	Follow-up	Stroke or systemic embolic event	Ischaemic stroke	Intracranial haemorrhage	Major haemorrhage
ARISTOTLE (2011)^149^ (*N* = 18 201; 25% with DM)	Patients with AF CHADS_2_ >1	Apixaban 5 mg b.i.d. Apixaban 2.5 mg b.i.d. in age >80 years, body weight <60 kg, creatinine >1.5 mg/mL	Warfarin (keep INR 2.0–3.0)	2 years	**1.27% vs. 1.60%** **HR 0.79 (0.66–0.95)**	0.97% vs. 1.05% HR 0.92 (0.74–1.13)	**0.33% vs. 0.80%** **HR 0.42 (0.30–0.58)**	**2.13% vs. 3.09%** **HR 0.69 (0.60–0.80)**
ARISTOTLE (2015)^153^ DM subgroup (*N* = 4547)	DM patients were younger, more CAD, higher CHADS_2_ and HAS-BLED				1.39% vs. 1.86% HR 0.75 (0.53–1.05)	Not reported	**0.34% vs. 0.70%** **HR 0.49 (0.25–0.95)**	3.01% vs. 3.13% HR 0.96 (0.74–1.25)
RE-LY (2009)^148^ (*N* = 18 113; 23.3% with DM)	Patients with AF CHADS_2_ >1 or CHA_2_DS_2_-VASc >2 for men or >3 for women	Dabigatran 110 mg b.i.d.	Warfarin (keep INR 2.0–3.0)	2 years	1.53% vs. 1.69% HR 0.91 (0.74–1.11)	1.34% vs. 1.20% HR 1.11 (0.89–1.40)	**0.23% vs. 0.74%** **HR 0.31 (0.20–0.47)**	**2.71% vs. 3.36%** **HR 0.80 (0.69–0.93)**
		Dabigatran 150 mg b.i.d.			**1.11% vs. 1.69%** **HR 0.66 (0.53–0.82)**	**0.92% vs. 1.20%** **HR 0.76 (0.60–0.98)**	**0.3% vs. 0.74%** **HR 0.40 (0.27–0.60)**	3.11% vs. 3.36% HR 0.93 (0.81–1.07)
RE-LY (2015)^152^ DM subgroup (*N* = 4221)	DM patients were younger, more CAD and PAD, higher CHA_2_DS_2_VASc scores	Dabigatran 110 mg b.i.d.	Warfarin (keep INR 2.0–3.0)		1.76% vs. 2.35% HR 0.74 (0.51–1.07)	1.62% vs. 1.65% HR 0.97 (0.64–1.40)	**0.22% vs. 0.81%** **HR 0.26 (0.11–0.65)**	3.81% vs. 4.19% HR 0.91 (0.70–1.19)
		Dabigatran 150 mg b.i.d.			**1.46% vs. 2.35%** **HR 0.61 (0.41–0.91)**	1.28% vs. 1.65% HR 0.76 (0.49–1.19)	0.47% vs. 0.81% HR 0.58 (0.29–1.16)	4.66% vs. 4.19% HR 1.12 (0.87–1.44)
ROCKET AF (2011)^150^ (*N* = 14 264; 39.9% with DM)	Patients with AF CHADS_2_ >2	Rivaroxaban 20 mg/day (15 mg/day if creatinine clearance 30–49 mL/min)	Warfarin (keep INR 2.0–3.0)	1.9 years	1.7% vs. 2.2% HR 0.79 (0.66–0.96)	2.11% vs. 2.27% HR 0.94 (0.75–1.17)	**0.8% vs. 1.2%** **HR 0.67 (0.47–0.93)**	5.6% vs. 5.4% HR 1.04 (0.90–1.20)
ROCKET AF (2015)^154^ DM subgroup (*N* = 5695)	DM patients were younger, more obese, higher BP, similar CHADS_2_ scores				1.7% vs. 2.1% HR 0.82 (0.63–1.08)	1.35% vs. 1.45% HR 0.94 (0.69–1.30)	0.5% vs. 0.8% HR 0.62 (0.36–1.05)	3.8% vs. 3.9% HR 1.00 (0.81–1.24)
ENGAGE AF-TIMI 48 (2013)^151^ (*N* = 21 105; 36.1% with DM)	Patients with AF CHADS_2_ >2	Edoxaban 30 mg/day (15 mg/day if creatinine clearance 30–50 mL/min, body weight <60 kg, or concomitant use of verapamil or quinidine)	Warfarin (keep INR 2.0–3.0)	2.8 years	1.61% vs. 1.50% HR 1.07 (0.87–1.31)	1.77% vs. 1.25% HR 1.41 (1.19–1.67)	**0.26% vs. 0.85%** **HR 0.30 (0.21–0.43)**	**1.61% vs. 3.43%** **HR 0.47 (0.41–0.55)**
		Edoxaban 60 mg/day (30 mg/day if creatinine clearance 30–50 mL/min, body weight <60 kg, or concomitant use of verapamil or quinidine)			**1.18% vs. 1.50%** **HR 0.79 (0.63–0.99)**	1.25% vs. 1.25% HR 1.00 (0.83–1.19)	**0.39% vs. 0.85%** **HR 0.47 (0.34–0.63)**	**2.75% vs. 3.43%** **HR 0.80 (0.71–0.91)**

Antiplatelet platelet and anticoagulant for secondary prevention in atrial fibrillation patients ineligible for vitamin K antagonist								
Study	Patients	Intervention	Control	Follow-up	Stroke, systemic emboli, MI, CVD death	Ischaemic stroke	Intracranial haemorrhage	Major haemorrhage
AVERROES (2011)^155^ (*N* = 5599; 19.6% with DM)	Patients with AF Ineligible for VKA CHADS_2_ >1, or documented PAD	Apixaban 5 mg b.i.d. Apixaban 2.5 mg b.i.d. in age >80 years, body weight <60 kg, creatinine >1.5 mg/mL	Aspirin 81–324 mg/day	1.1 years	**4.2% vs. 6.4%** **HR 0.66 (0.53–0.83)**	**1.1% vs. 3.0%** **HR 0.37 (0.25–0.55)**	0.4% vs. 0.4% HR 0.85 (0.38–1.90)	1.4% vs. 1.2% HR 1.13 (0.74–1.75)

Antiplatelet and anticoagulant studies for secondary prevention in individuals with cerebrovascular disease.

Significant differences are highlighted in bold.

b.i.d., twice daily; CAD, coronary artery disease; CHA_2_DS_2_-VASc, score, Congestive Heart failure, hypertension, Age ≥75 (doubled), Diabetes, Stroke (doubled), Vascular disease, Age 65–74, and Sex (female); CHADS_2_ score, Cardiac failure, Hypertension, Age, Diabetes, Stroke (doubled); CVD, cardiovascular disease; DM, diabetes mellitus; HAS-BLED score, hypertension, abnormal renal/liver function (1 point each), stroke, bleeding history or predisposition, labile INR, elderly (65 years), drugs/alcohol concomitantly (1 point each); HR, hazard ratio; MI, myocardial infarction; mRS, modified Rankin scale; NIHSS, National Institutes of Health Stroke Scale; PAD, peripheral artery disease; TIA, transient ischaemic stroke; VKA, vitamin K antagonist.

### Long-term non-cardioembolic stroke prevention

The effect of aspirin on secondary prevention in stroke/TIA patients is well-established, as are the effects of clopidogrel and aspirin/dipyridamole combination.^138^ The benefits of aspirin (75–150 mg daily) appear to be most pronounced in the first 6–12 weeks following the event, while combination with dipyridamole may offer better longer-term protection.^139^ Clopidogrel is as good as aspirin/dipyridamole combination and is preferred for secondary prevention, particularly with the frequent headaches with aspirin/dipyridamole combination leading to discontinuation.^140^ ^,^ ^141^

DAPT for long-term use is discouraged as studies showed excessive bleeding without a vascular benefit.^142–145^

Taken together, for the management of DM individuals, aspirin is justified 24 h after an acute event requiring reperfusion therapy followed by a switch to clopidogrel 3 months later (or continue aspirin while adding dipyridamole). In those not receiving reperfusion therapy, DAPT can be immediately started (provided haemorrhagic stroke is ruled out) and continued for 21 days followed by long-term monotherapy with clopidogrel (or a combination of aspirin/dipyridamole), which applies to individuals with and without diabetes (*Figure 2*).^139–141^

### Stroke prevention in association with non-valvular atrial fibrillation

As discussed above, anticoagulation is recommended for those with AF and elevated CHA_2_DS_2_-VASc score, of which DM is a component.^113^ ^,^ ^146^ ^,^ ^147^

Potential superior efficacy of NOACs, together with reduced bleeding events and reduced need for monitoring, offers distinct advantages over VKAs. An analysis of four large RCTs [RE-LY, ARISTOTLE, ROCKET-AF, and ENGAGE AF-TIMI 48 (23%, 25%, 40%, and 36% with DM, respectively)],^148–151^ enrolling over 70 000 patients with non-valvular AF with at least one additional risk factor for stroke, demonstrated that dabigatran or factor Xa inhibitors (apixaban, rivaroxaban, edoxaban) are at least as efficacious as warfarin, and in some cases superior, in preventing stroke, whilst reducing bleeding risk. Sub-analysis of DM patients in these trials showed similar anti-thrombotic benefits of NOACs but bleeding risk reduction appeared to be attenuated.^152–154^

In AF patients who are ineligible for VKA, apixaban (AVERROES trial; 20% with diabetes) is the only NOAC that has demonstrated superior efficacy to aspirin in preventing MACE (stroke, systemic embolism, MI, and vascular death) with similar bleeding risk in the whole study population with no subgroup analysis conducted for those with DM.^155^

In summary, current evidence supports OAC in DM individuals with AF with or without a history of stroke who fulfil treatment criteria and do not have excessive bleeding risk. NOACs are preferable to VKAs in eligible patients.

Timing of initiation (or therapy resumption) of OAC in AF patients suffering an acute stroke is a challenging area. Based largely on two studies, RAF and RAF-NOACs,^156^ ^,^ ^157^ the American Heart Association/American Stroke Association 2018/2019 guidelines recommend starting OAC within 4–14 days of an acute ischaemic stroke.^132^ The European Heart Rhythm Association-ESC guidelines give a more structured recommendation with the ‘1–3–6–12 days rule’.^158^ In brief, OAC should be initiated or reinstated after 1 day for TIA, 3 days for mild stroke (NIHSS score <8), 6 days for moderate stroke (NIHSS score 8–15), and 12 days for severe stroke (NIHSS score ≥16). These recommendations are based solely on expert consensus without robust RCTs supporting this approach. Of note, bridging with full-dose low-molecular-weight heparin before or together with VKA is not recommended.^159^  *Table 4* summarizes key studies on antithrombotic agents in cerebrovascular disease.

## Conclusions and future directions

While a large number of studies investigated the best antithrombotic strategy in vascular disease patients, there is still a distinct lack of DM-specific RCTs, particularly for secondary prevention. The heterogeneous vascular risk in DM patients, which can vary in the same individual according to DM duration and development of complications, adds to the complexity and prevents guidelines from making concrete recommendations. For example, advanced renal disease may alter both thrombosis and bleeding risk and may even limit the use of some antithrombotic therapies.^33^ The increased weight in DM individuals may also affect the response to antithrombotic agents, reviewed elsewhere.^160^ A key difficulty remains the lack of biomarker(s) that accurately predicts thrombotic/bleeding risk and response to therapy.

Given current knowledge, primary prevention with antiplatelet agents, mainly aspirin, may only be considered in higher-risk individuals. Following ACS, DAPT is necessary using aspirin and ticagrelor or prasugrel, usually for 12 months but also longer term with aspirin and ticagrelor in high thrombotic risk patients. In stable atherosclerotic disease, the combination of aspirin and very-low-dose rivaroxaban is useful, particularly in the presence of PAD (*Figure 2*).^161^ In individuals with stroke, the choice of antithrombotic therapy is dictated by whether the individual required reperfusion and the presence of AF (*Figure 2* and *Graphical Abstract*).

Areas for future research include the development of reliable biomarkers and/or *in silico* model, able to assess thrombotic risk and response to therapy. Moreover, DM-specific studies are warranted rather than subgroup, and often *post hoc*, analyses of cardiovascular trials designed for the wider population (DM-specific studies are summarized in Supplementary material online, *Table S3* and gaps in knowledge/future work in Supplementary material online, *Table S4*). This will require greater collaboration between metabolic and vascular medical disciplines to design appropriate studies aiming to reduce vascular events and improve clinical outcomes in the high-risk DM population.

## Supplementary material

Supplementary material is available at *European Heart Journal* online.

## Funding

N.K. is funded by Faculty of Medicine, Prince of Songkla University, Thailand. Research work in Ajjan’s laboratory is funded by the National Institute for Health Research, Diabetes UK, British Heart Foundation, Biotechnology and Biological Sciences Research Council, Abbott Diabetes Care and Avacta Life Sciences. 

**Conflict of interest:** R.A.A. reports grants, personal fees and other from Abbott Diabetes Care, personal fees from AstraZeneca, personal fees from Boehringer Ingelheim, personal fees from Eli Lilly, personal fees from Menarini Pharmaceuticals, personal fees from Novo Nordisk, outside the submitted work; N.K. has nothing to disclose; L.B. reports grants from AstraZeneca, the European Union-IMI and -H2020, Carlos III Institute of Health-Spain, and CIBERCV-Spain; personal fees (scientific advisory boards/speaker fees) from AstraZeneca, Lilly, BMS/Pfizer, SANOFI, BAYER, International Aspirin Foundation, Glycardial SL, PACE and FICYE, all outside the submitted work; G.V. reports grants from AstraZeneca, outside the submitted work; D.A.G. reports personal fees and other from Astra Zeneca, grants from Bayer, personal fees from Boehringer Ingelheim, outside the submitted work; D.J.A. reports grants and personal fees from Amgen, grants and personal fees from Aralez, grants and personal fees from Bayer, grants and personal fees from Biosensors, grants and personal fees from Boehringer Ingelheim, grants and personal fees from Bristol-Myers Squibb, grants and personal fees from Chiesi, grants and personal fees from Daiichi-Sankyo, grants and personal fees from Eli Lilly, personal fees from Haemonetics, grants and personal fees from Janssen, grants and personal fees from Merck, personal fees from PhaseBio, personal fees from PLx Pharma, personal fees from Pfizer, grants and personal fees from Sanofi, personal fees from the Medicines company, grants and personal fees from CeloNova, personal fees from St Jude Medical, grants from CSL Behring, grants from Eisai, grants from Gilead, grants from Idorsia Pharmaceuticals Ltd, grants from Matsutani Chemical Industry Co., grants from Novartis, grants from Osprey Medical, grants from Renal Guard Solutions, grants from Scott R. MacKenzie Foundation, grants and personal fees from Astra Zeneca, outside the submitted work; D.R. has nothing to disclose; B.R. reports personal fees from Novartis, personal fees from Medscape, grants from Bayer AG, grants from Italian Medicines Agency, outside the submitted work; R.F.S. reports personal fees from Bayer, personal fees from Bristol-Myers Squibb/Pfizer, grants and personal fees from AstraZeneca, grants and personal fees from Thromboserin, personal fees from Haemonetics, personal fees from Amgen, grants and personal fees from Glycardial Diagnostics, personal fees from Portola, personal fees from Medscape, grants and personal fees from Cytosorbents, personal fees from Intas Pharmaceuticals, personal fees from Hengrui, personal fees from Sanofi Aventis, personal fees from Idorsia, personal fees from PhaseBio, outside the submitted work.

## Supplementary Material

ehab128_Supplementary_Data
