# Supplementary material for: Antithrombotic therapy in diabetes: which, when, and for how long?
Source: Eur Heart J. 2021 Mar 25;42(23):2235–59. doi: 10.1093/eurheartj/ehab128 (PMC8203081; doi:10.1093/eurheartj/ehab128)
Supplement: ehab128_Supplementary_Data [file ehab128_supplementary_data.zip › ehab128-supl_data/suppl Figure 1-R1.pptx]

## Slide 1
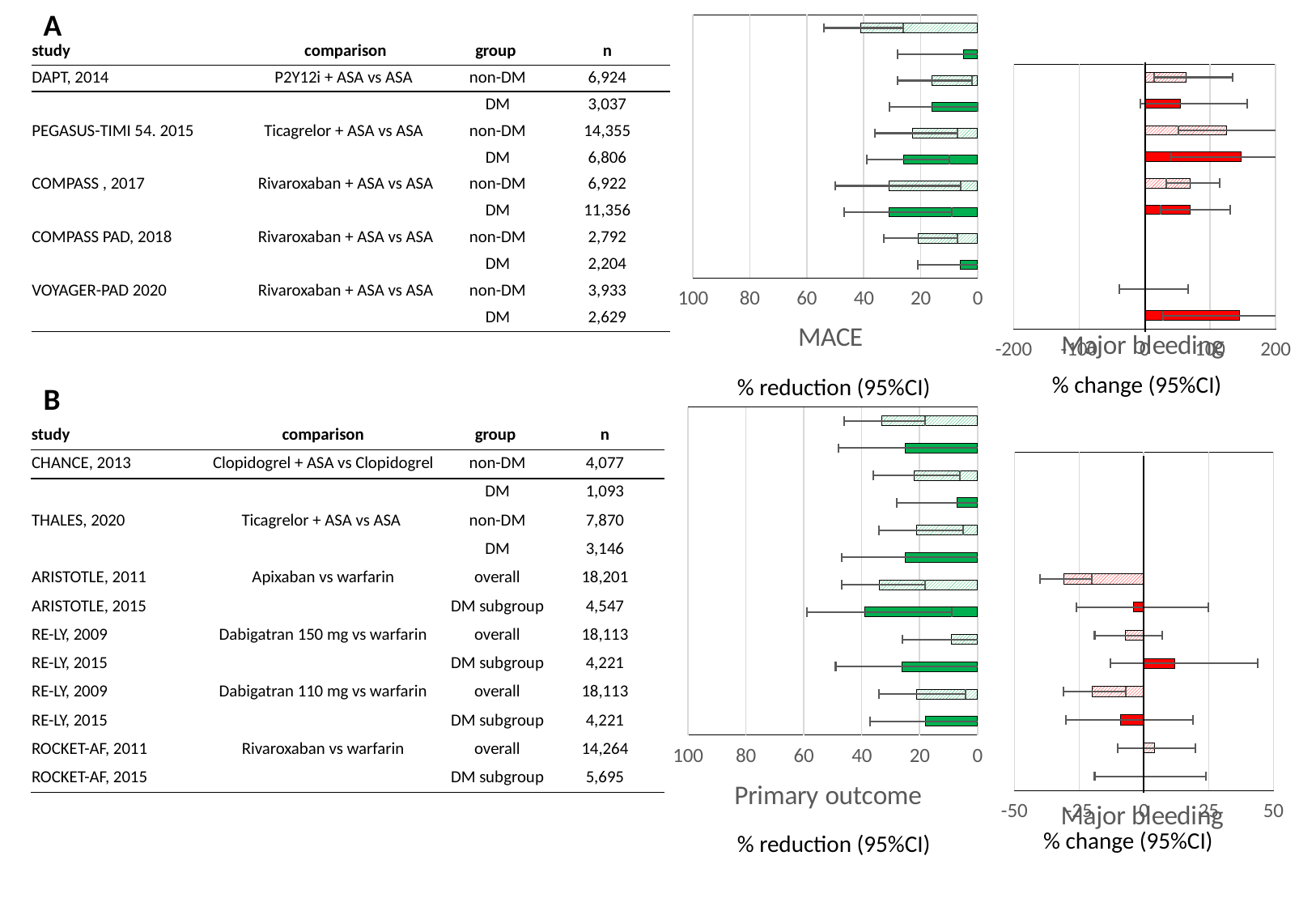

A
### Chart
| Category | |
|---|---|
### Chart
| Category | |
|---|---|
### Chart
| Category | |
|---|---|
### Chart
| Category | |
|---|---|% change (95%CI)
| study | comparison | group | n |
| --- | --- | --- | --- |
| DAPT, 2014 | P2Y12i + ASA vs ASA | non-DM | 6,924 |
| | | DM | 3,037 |
| PEGASUS-TIMI 54. 2015 | Ticagrelor + ASA vs ASA | non-DM | 14,355 |
| | | DM | 6,806 |
| COMPASS , 2017 | Rivaroxaban + ASA vs ASA | non-DM | 6,922 |
| | | DM | 11,356 |
| COMPASS PAD, 2018 | Rivaroxaban + ASA vs ASA | non-DM | 2,792 |
| | | DM | 2,204 |
| VOYAGER-PAD 2020 | Rivaroxaban + ASA vs ASA | non-DM | 3,933 |
| | | DM | 2,629 |
% change (95%CI)
% reduction (95%CI)
B
| study | comparison | group | n |
| --- | --- | --- | --- |
| CHANCE, 2013 | Clopidogrel + ASA vs Clopidogrel | non-DM | 4,077 |
| | | DM | 1,093 |
| THALES, 2020 | Ticagrelor + ASA vs ASA | non-DM | 7,870 |
| | | DM | 3,146 |
| ARISTOTLE, 2011 | Apixaban vs warfarin | overall | 18,201 |
| ARISTOTLE, 2015 | | DM subgroup | 4,547 |
| RE-LY, 2009 | Dabigatran 150 mg vs warfarin | overall | 18,113 |
| RE-LY, 2015 | | DM subgroup | 4,221 |
| RE-LY, 2009 | Dabigatran 110 mg vs warfarin | overall | 18,113 |
| RE-LY, 2015 | | DM subgroup | 4,221 |
| ROCKET-AF, 2011 | Rivaroxaban vs warfarin | overall | 14,264 |
| ROCKET-AF, 2015 | | DM subgroup | 5,695 |
% reduction (95%CI)
