# Supplementary material for: Antithrombotic therapy in diabetes: which, when, and for how long?
Source: Eur Heart J. 2021 Mar 25;42(23):2235–59. doi: 10.1093/eurheartj/ehab128 (PMC8203081; doi:10.1093/eurheartj/ehab128)
Supplement: ehab128_Supplementary_Data [file ehab128_supplementary_data.zip › ehab128-supl_data/Supplementary S1-R1-RA.docx]

**Supplementary S1**

**Search strategy**

To avoid bias, appropriate searches were conducted for each section, focused on publications in the past 10 years. After receiving the various sections from co-authors, an additional PubMed search was conducted by the first author that concentrated on clinical trials (including RCTs), meta-analysis and review articles (including systematic reviews) to ensure relevant studies are included. Abstracts were reviewed and relevant manuscripts were included in the review. Search terms used included:

Antiplatelet (or aspirin) AND primary prevention AND diabetes;

Antiplatelet (or antithrombotic or anticoagulant) AND secondary prevention AND diabetes;

Antiplatelet (or antithrombotic or anticoagulant) AND cardiovascular AND diabetes;

Antiplatelet (or antithrombotic or anticoagulant) AND coronary artery AND diabetes;

Antiplatelet (or antithrombotic or anticoagulant) AND cerebrovascular AND diabetes;

Antiplatelet (or antithrombotic or anticoagulant) AND peripheral vascular AND diabetes;

Aspirin (or P2Y12, prasugrel, ticagrelor, rivaroxaban, apixaban, dabigatran or warfarin) AND cardiovascular AND diabetes.

**Long term therapy for secondary cardiovascular protection - vorapaxar**

The TRA 2°P-TIMI 50 study, including individuals with a history of atherosclerosis (n=26,449), showed that vorapaxar (2.5mg od) added to aspirin and/or a thienopyridine significantly reduced MACE [9.3% vs 10.5%, RRR 13% (0.80-0.94); p<0.001] but with 66% increase in bleeding complications [4.2% vs 2.5%, HR 1.66 (1.43-1.93); p<0.001], and a 2-fold increase in intracranial haemorrhages (p<0.001) with a NNH/NNT ratio <1. A similar pattern was evident in the DM subgroup (p=0.61 for interaction) and when individuals with a history of MI were analysed.[^95^](#_ENREF_95)^,^ [^96^](#_ENREF_96) Unexpectedly, those with diabetes and previous MI demonstrated a possible net clinical benefit (combined outcome of CV death, MI, stroke, recurrent ischemia and moderate/severe bleeding) [16.4% vs 19.6%; HR 0.79 (0.67–0.93); p=0.005], in contrast to individuals without DM [10.4% vs 10.9%; HR 0.95 (0.85–1.06); p=0.32].[^97^](#_ENREF_97) However, we should be cautious in interpreting *post hoc* analyses and, given the absence of a clear overall benefit in main study group (due to high bleeding complications), clinical use of this agent remains very limited.

**DM and atrial fibrillation**

Whilst the precise pathophysiological mechanisms linking DM with AF are not fully understood, hyperglycaemia and glycaemic fluctuations appear to contribute.[^101-104^](#_ENREF_101) Moreover, elevated basal HbA1c levels are associated with a higher recurrence rate of AF after catheter ablation.[^105^](#_ENREF_105) There is no clear evidence that intensive glycaemic control reduces AF risk, but prospective trials to specifically address this are lacking. Cohort studies and observations from RCTs suggest that hypoglycaemic agents such as metformin and thiazolidinediones are associated with lower AF risk by unknown mechanisms but avoidance of hypoglycaemia may have a role.[^106-108^](#_ENREF_106) Recent data from the DECLARE-TIMI 58 study (17,160 patients with DM) has shown that dapagliflozin reduced incidence of AF or atrial flutter [7.8 vs 9.6 events per 1000 patient-years, HR 0.81 (0.68 to 0.95), p=0.009] by mechanisms that are not entirely clear.[^109^](#_ENREF_109)

**Symptomatic PAD - vorapaxar**

The TRA 2°P-TIMI 50 trial showed no difference in MACE in a subgroup of 3,787 patients with symptomatic PAD (35.8% with DM) treated by voraxapar versus placebo.^123^ However, it did demonstrate a reduction in hospitalisation for acute limb ischaemia (2.3% versus 3.9%, HR 0.58 (0.39–0.86); p=0.006] and peripheral artery revascularization [18.4% vs 22.2%, HR 0.84 (0.73– 0.97); p=0.017] with voraxapar, but with an increased bleeding risk [7.4% vs 4.5%, HR 1.62 (1.21–2.18); p=0.001]. As discussed previously, the lack of benefit in a non-DM cohort and the high bleeding risk, including intracerebral haemorrhage, has limited adoption of this agent in clinical practice.

.
