# Supplementary material for: Antithrombotic therapy in diabetes: which, when, and for how long?
Source: Eur Heart J. 2021 Mar 25;42(23):2235–59. doi: 10.1093/eurheartj/ehab128 (PMC8203081; doi:10.1093/eurheartj/ehab128)
Supplement: ehab128_Supplementary_Data [file ehab128_supplementary_data.zip › ehab128-supl_data/supplementary table 1_R1.docx]

**Supplementary table 1. Summary of the agents used following an acute coronary event**

|  | **Aspirin** | **Clopidogrel** | **Ticagrelor** | **Prasugrel** | **Cangrelor** | **Tirofiban** | **Eptifibatide** | **Abciximab** | **Rivaroxaban** |
| --- | --- | --- | --- | --- | --- | --- | --- | --- | --- |
| Site of action | Inhibitor of clyclooxygenase | Thienopyridine, binds to P2Y_12_ receptor | Cyclopentile-triazolopyrimidine, binds to P2Y_12_ receptor | Thienopyridine, binds to P2Y_12_ receptor | Adenosine triphosphate analogue, binds to P2Y_12_ receptor | Glycoprotein IIb/IIIa receptor inhibitor | Glycoprotein IIb/IIIa receptor inhibitor | Glycoprotein IIb/IIIa receptor inhibitor | Factor Xa inhibitor |
| Administration | Oral  Once daily | Oral  Once daily | Oral  Twice daily | Oral  Once daily | Intravenous | Intravenous | Intravenous | Intravenous | Oral  Twice daily |
| Onset of action | 1-2 h | 2-6 h | 30 min | 30 min | 2 min | Minutes | Minutes | Minutes | 2.5-4h |
| Half-life (active metabolite) | 15-20 min | 30-60 min | 7-9 h | 30-60 min (distribution)  2-15h (elimination) | 3-6 min | 1.2-2 h | 2.5-2.8 h | 10-30 min | 5-9h |
| Binding reversibility | Irreversible | Irreversible | Reversible | Irreversible | Reversible | Reversible | Reversible | Irreversible | Reversible |
| Route of elimination | Renal | Renal 50%  Biliary 46% | Hepatic | Renal 68% | 58% renal  Biliary 35% | Renal (30-60%)  Biliary (40-70%) | Renal 50% | Plasma proteases | Renal 60% |
| Offset after discontinuation | 5 days | 5-7 days | 3-5 days | 7-10 days | 30-60 min | 4 h | 4 h | 12 h | 11 h |
