# Supplementary material for: Antithrombotic therapy in diabetes: which, when, and for how long?
Source: Eur Heart J. 2021 Mar 25;42(23):2235–59. doi: 10.1093/eurheartj/ehab128 (PMC8203081; doi:10.1093/eurheartj/ehab128)
Supplement: ehab128_Supplementary_Data [file ehab128_supplementary_data.zip › ehab128-supl_data/supplementary table 2_R1.docx]

**Supplementary Table 2: Guidelines on the use of low-dose aspirin in primary prevention**

| **Organization (year)** | **Recommendation** | **Class**  **(Level of evidence)** |
| --- | --- | --- |
| ESC (2019)[^51^](#_ENREF_51) | In patients with DM at high*/very high** risk, aspirin (75–100 mg/day) may be considered in primary prevention in the absence of clear contraindications^†^. | IIb (A) |
| ESC (2019)[^51^](#_ENREF_51) | When low-dose aspirin is used, proton pump inhibitors should be considered to prevent gastrointestinal bleeding. | IIa (A) |
| AHA/ADA (2019)[^52^](#_ENREF_52) | Aspirin therapy (75–162mg/day) may be considered as a primary prevention strategy in those with diabetes who are at increased cardiovascular risk, after a discussion with the patient on the benefits versus increased risk of bleeding. | C |
| USPSTF (2016)[^53^](#_ENREF_53) | The USPSTF recommends initiating low-dose aspirin use for the primary prevention of cardiovascular disease (CVD) and colorectal cancer (CRC) in adults aged 50 to 59 years who have a 10% or greater 10-year CVD risk, are not at increased risk for bleeding, have a life expectancy of at least 10 years, and are willing to take low-dose aspirin daily for at least 10 years. | B |
| USPSTF (2016)[^53^](#_ENREF_53) | The decision to initiate low-dose aspirin use for the primary prevention of CVD and CRC in adults aged 60 to 69 years who have a 10% or greater 10-year CVD risk should be an individual one. Persons who are not at increased risk for bleeding, have a life expectancy of at least 10 years, and are willing to take low-dose aspirin daily for at least 10 years are more likely to benefit. Persons who place a higher value on the potential benefits than the potential harms may choose to initiate low-dose aspirin. | C |
| CCS (2011)[^54^](#_ENREF_54) | For patients with diabetes and aged >40 years and at low risk for major bleeding, low-dose ASA (75-162 mg daily) may be considered for primary prevention in patients with other cardiovascular risk factors for which its benefits are established. | IIb (B) |

Guidelines are listed in descending order of publication.

* Patients with target organ damage (proteinuria, renal impairment defined as eGFR≥30mL/min/1.73m^2^), **or** three or more major risk factors (age, hypertension, dyslipidemia, smoking, obesity), **or** early onset T1DM of long duration (>20 years).

** Patients with DM duration ≥10 years without target organ damage plus any other additional risk factor

^†^ defined as: gastrointestinal bleeding, peptic ulceration within the previous 6 months, active hepatic disease, or history of aspirin allergy.

Abbreviations: ADA: American Diabetes Association; AHA: American Heart Association; CRC: colorectal cancer; CVD: cardiovascular disease; DM: diabetes mellitus; ESC: European Society of Cardiology; MI: myocardial infarction; USPSTF: United States Preventive Services Task Force; CCS: Canadian Cardiovascular Society.
