# Supplementary material for: Antithrombotic therapy in diabetes: which, when, and for how long?
Source: Eur Heart J. 2021 Mar 25;42(23):2235–59. doi: 10.1093/eurheartj/ehab128 (PMC8203081; doi:10.1093/eurheartj/ehab128)
Supplement: ehab128_Supplementary_Data [file ehab128_supplementary_data.zip › ehab128-supl_data/supplementary table 3_R1.docx]

**Supplementary Table 3: A. Summary of primary prevention studies specifically dedicated to address antithrombotic therapy in patients with diabetes.**

| **Study** | **Patient** | **Primary efficacy endpoint** | **median follow-up** | **Predicted vs observed incidence and expected benefit** | **Absolute and relative benefit** | **Absolute and relative harm** | **Comments** |
| --- | --- | --- | --- | --- | --- | --- | --- |
| JPAD (2008)[^43^](#_ENREF_43) | **2,539** T2D patients  without a history of atherosclerotic disease | Sudden death; death from coronary, cerebrovascular, and aortic causes; nonfatal acute MI; UA; exertional angina; nonfatal ischaemic and haemorrhagic stroke; TIA; or nonfatal aortic and PVD | 4.4 years | Predicted: 5.2%/yr  vs  Observed: 1.7%/yr  Expected benefit:  30% RRR | Aspirin: 5.4%  Placebo: 6.7%  HR: 0.80 (0.58-1.10) | Any GI bleeding:  Aspirin: n=12  Placebo: n=4 | Observed primary endpoint rate was ≈1/3 of predicted.  The expected benefit likely unrealistic based on previous data (trial largely underpowered) |
| POPADAD (2008)[^44^](#_ENREF_44) | **1,276** adults aged ≥40 with T1D or T2D and ABI≤0.99 (asymptomatic) | Death from CAD or stroke, non-fatal MI or stroke, or amputation for critical limb ischaemia; and death from CAD or stroke. | 6.7 years | Predicted: 28%/yr  vs  observed: 2.9%/yr  Expected benefit:  25% RRR | Aspirin: 18.2%  Placebo: 18.3%  HR: 0.98 (0.76 to 1.26) | Any GI bleeding  Aspirin: 4.4%  Placebo: 4.9%  HR: 0.90 (0.53 to 1.52) | Observed events were approx. 1/10 of predicted.  The expected benefit was likely unrealistic based on previous data (trial was largely underpowered) |
| ASCEND (2018)[^45^](#_ENREF_45) | **15,480** Patients aged ≥40 years with DM and no evident CV disease | Non-fatal MI, non-fatal stroke (excluding confirmed ICH), TIA or death from any vascular cause (excluding confirmed ICH) | 7.4 years | Predicted:  1.2-1.3%/yr  vs observed: 1.3%/yr  Expected benefit:  15% RRR | **Aspirin: 8.5%**  **Placebo: 9.6%**  **HR: 0.88 (0.79-0.97)** | BARC 2,3 and 5 bleeding  **Aspirin: 4.1%**  **Placebo 3.2%**  **HR: 0.88 (0.79-0.97)**  ICH  Aspirin: 0.7%  Placebo: 0.6%  HR 1.22 (0.82-1.81)  Fatal bleeding  Aspirin: 0.2%  Placebo: 0.2%  HR 1.18 (0.61-2.30) | Consistency between predicted and observed incidence event rate  NNT/NNH: 0.81 |
| THEMIS (2019)[^47^](#_ENREF_47) | **19,220** patients with DM, ≥50 years, stable CAD with no previous MI or stroke  Randomised to ticagrelor or placebo on a background of aspirin therapy | Stroke, MI and CV death | 3.3 years | Predicted benefit: 16% RRR  Predicted: 2.5%/yr  vs  observed:2.5%/yr | **Ticagrelor: 7.7%**  **Placebo: 8.5%**  **HR: 0.90 (0.81-0.99)** | TIMI major bleeding  **Ticagrelor: 2.2%**  **Placebo: 1%**  **HR: 2.32 (1.82-2.94)**  BARC 3-5 bleeding:  **Ticagrelor: 3.7%**  **Placebo: 1.7%**  **HR: 2.36 (1.96–2.84**)  ICH:  **Ticagrelor: 0.7%**  **Placebo: 0.5%**  **HR: 1.71 (1.18-2.48)** | High rate of ticagrelor discontinuation:  Placebo 25% vs Ticagrelor: 35%  HR 1.50 (1.42-1.58)  Predicted benefit higher than observed.  NNT/NNH: 1.48 (TIMI-major defined bleeding) |

Abbreviations: ABI: ankle brachial index; BARC: Bleeding Academic Research Consortium; CAD: coronary artery disease; CI: confidential interval; CV: cardiovascular; DM: diabetes mellitus; GI: gastrointestinal; ICH: intracranial hemorrhage; HR: hazard ratio; MI: myocardial infarction; NA: not applicable; NNH: number needed to harm; NNT: number needed to treat; RRR: relative risk reduction; TIMI: Thrombolysis in Myocardial Infarction; UA: unstable angina; TIA: transient ischemic attack; PVD: peripheral vascular disease. Significant differences and HR are reported in bold character.
