# Supplementary material for: Antithrombotic therapy in diabetes: which, when, and for how long?
Source: Eur Heart J. 2021 Mar 25;42(23):2235–59. doi: 10.1093/eurheartj/ehab128 (PMC8203081; doi:10.1093/eurheartj/ehab128)
Supplement: ehab128_Supplementary_Data [file ehab128_supplementary_data.zip › ehab128-supl_data/supplementary table 4-R1.docx]

**Supplementary Table 4: Gaps in knowledge in relation to antithrombotic therapy in diabetes and future work.**

| **Gap in knowledge** | **Studies required** |
| --- | --- |
| **Lack of a reliable test for thrombosis risk and response to therapy** | Development of appropriate platelet function and fibrin network tests that can accurately assess thrombosis risk |
| **Targeting alternative thrombosis factors** | Platelets: 5HT_2A_ receptor antagonists, PAR-4 antagonists, GPVI antagonists  Coagulation factors: TAFI, PI, PAI-1, Factor XI, Factor XII |
| **Primary prevention** | Role of different aspirin dosing (e.g. twice daily low dose)  Use of antithrombotic agent other than aspirin for PP  Separate studies on individuals with variable CV risk |
| **Secondary prevention in coronary artery disease** | Different antithrombotic strategies following an acute event (different DAPT or antiplatelet/anticoagulant combination)  Long-term antithrombotic monotherapy with different antithrombotic agents (DAPT or antiplatelet/anticoagulant combination), including characterisation of the subgroups requiring more aggressive antithrombotic regimen |
| **Secondary prevention in peripheral artery disease** | Diabetes-specific studies to determine the best antithrombotic strategy for the management of symptomatic disease |
| **Secondary prevention in cerebrovascular disease** | Diabetes-specific studies to establish the best antithrombotic strategy following an acute event  Diabetes-specific studies to establish long-term antithrombotic strategy both in the presence of sinus rhythm and in those with cardiac arrhythmias |

HT: Hydroxytryptamine, PAR: protease activated receptor, GP: glycoprotein, TAFI: Thrombin activatable firbinolysis inhibitor, PI: plasmin inhibitor, PAI-1: plasminogen activator inhibitor, DAPT: dual antiplatelet therapy.
